# Sperm-binding regions on bovine egg zona pellucida glycoprotein ZP4 studied in a solid supported form on plastic plate

**DOI:** 10.1371/journal.pone.0254234

**Published:** 2021-07-09

**Authors:** Kamila Dilimulati, Misaki Orita, Ganbat Undram, Naoto Yonezawa

**Affiliations:** Department of Chemistry, Graduate School of Science, Chiba University, Chiba, Japan; Bryant University, UNITED STATES

## Abstract

The zona pellucida (ZP) is a transparent envelope that surrounds the mammalian oocyte and mediates species-selective sperm–oocyte interactions. The bovine ZP consists of the glycoproteins ZP2, ZP3, and ZP4. Sperm-binding mechanisms of the bovine ZP are not yet fully elucidated. In a previous report, we established the expression system of bovine ZP glycoproteins using Sf9 insect cells and found that the ZP3/ZP4 heterocomplex inhibits the binding of sperm to the ZP in a competitive inhibition assay, while ZP2, ZP3, ZP4, the ZP2/ZP3 complex, and the ZP2/ZP4 complex do not exhibit this activity. Here, we show that bovine sperm binds to plastic plates coated with ZP4 in the absence of ZP3. We made a series of ZP4 deletion mutants to study the sperm-binding sites. The N-terminal region, Lys-25 to Asp-136, and the middle region, Ser-290 to Lys-340, of ZP4 exhibit sperm-binding activity. These results suggest that among the three components of bovine ZP glycoproteins, ZP4 contains the major potential sperm-binding sites, and the formation of a multivalent complex is necessary for the sperm-binding activity of ZP4.

## Introduction

**Fig 1:**
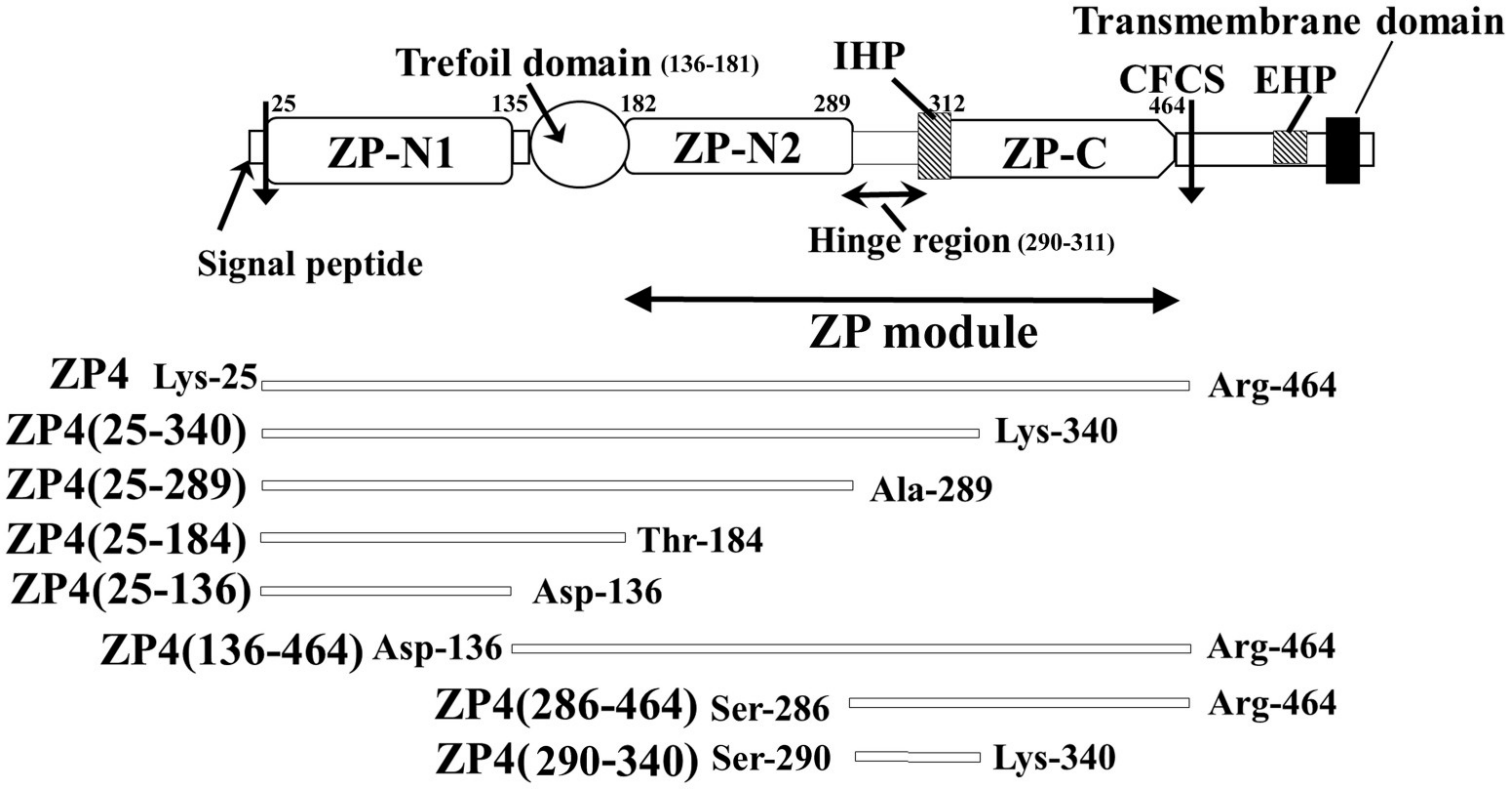
Schematic representation of the bovine zona pellucida (ZP) 4 polypeptide precursor and the recombinant ZP4 mutants examined in this study. The bovine ZP4 precursor is synthesized as a transmembrane protein consisting of a signal peptide, the N-terminal ZP-N-like domain (ZP-N1), the trefoil domain, the N-terminal half domain of the ZP module (ZP-N2), the flexible hinge region, an internal hydrophobic patch (IHP) in the C-terminal half domain of the ZP module (ZP-C), a consensus furin cleavage site (CFCS), an external hydrophobic patch (EHP), and a transmembrane domain. The mature ZP4 polypeptide includes Lys-25 to Arg-464. The ZP4 fragments examined in this study are shown by open bars. Translation initiation Met is numbered as 1.

Mammalian oocytes are surrounded by a transparent envelope called the zona pellucida (ZP). The ZP is involved in oocyte maturation, species-selective sperm recognition, blocking polyspermy and early embryo development [1–5]. The ZP comprises either three or four types of glycoproteins depending on the species. Many of mammalian species studied thus far including human possess four ZP glycoproteins (ZP1, ZP2, ZP3, and ZP4) [6–8], whereas bovine and porcine ZPs consist of three glycoproteins (ZP2, ZP3, and ZP4) [9]; mouse ZP also consists of three glycoproteins (ZP1, ZP2, and ZP3) [10]. All ZP proteins contain a bipartite module called the ZP module, which consists of ~260 amino acids and contains eight conserved Cys residues [11, 12]. The ZP modules of ZP glycoproteins are involved in the formation of ZP filaments [13] and consist of three regions: the ZP-N and ZP-C domains, and the flexible hinge region connecting the two domains [14] (see Fig 1). ZP glycoproteins are synthesized as precursor transmembrane proteins, transported to the plasma membrane, and processed at the consensus furin cleavage site (CFCS) [15]. The polymerization of ZP glycoprotein precursors are inhibited by an interaction between an external hydrophobic patch (EHP) located between the CFCS and the transmembrane domain and an internal hydrophobic patch (IHP) located at the beginning of the ZP-C domain [16] (see Fig 1). Upon cleavage at the CFCS and release of the ectodomain from the transmembrane domain, dissociation of IHP from EHP triggers ZP protein assembly [14].

The mechanisms of sperm–ZP interaction in mammals have been investigated for more than three decades. In mice and humans, sperm bind to the N-terminal domain of ZP2 as revealed *in vivo* by using a series of transgenic mice and also *in vitro* by using recombinant proteins [17, 18]. In pigs and cow, sperm bind to ZP3/ZP4 heterocomplex, though only *in vitro* experiments have been done [19, 20]. This discrepancy among species may be because of the differences in the ZP protein compositions. Bovine and porcine ZP do not have ZP1, while mouse and human ZP have ZP1. Thus, it is still necessary to investigate the mechanisms of sperm–ZP interaction in the mammalian species other than mice and humans to elucidate whether mammals have a common mechanism or not. In this study, we further studied the sperm-binding sites on bovine ZP.

Native bovine ZP glycoproteins obtained from ovaries show a broad band of an average apparent molecular mass of 74 kDa on sodium dodecyl sulfate-polyacrylamide gel electrophoresis (SDS-PAGE) under nonreducing conditions because of heterogeneity in the carbohydrate moieties [9]. Bovine ZP2, ZP3, and ZP4 show distinct bands of 72, 45, and 58 kDa, respectively, on SDS-PAGE under nonreducing conditions after removal of N-acetyllactosamine repeats at the nonreducing portion of carbohydrate chains by digestion with endo-β-galactosidase [9]. Among the three components partially purified from the endo-β-galactosidase digests, ZP4 contaminated with ZP3 exhibits the highest inhibition of sperm–ZP binding based on a competitive inhibition assay [21]. In previous studies on porcine ZP, however, a trace amount of ZP3 contamination in ZP4 is essential for inhibition of sperm–ZP binding [19, 22]. To obtain bovine ZP4 without contamination of ZP3, a baculovirus-Sf9-based cell expression system of bovine ZP glycoproteins was established [20]. Each of ZP2, ZP3 and ZP4 alone, the ZP2/ZP4 complex and the ZP2/ZP3 complex co-expressed in Sf9 cells do not inhibit sperm–ZP binding, whereas the ZP3/ZP4 complex and the ZP2/ZP3/ZP4 complex inhibit the binding [20]. Recently, it was reported that the N-terminal fragment of ZP3 (Arg-32 to Glu-178) interacts with ZP4 and the complex between the ZP3 fragment and ZP4 inhibits sperm–ZP binding [23].

Although ZP3/ZP4 complex formation is necessary for the inhibitory activity for bovine sperm–ZP binding, it is not yet clear whether sperm-binding sites are formed on the ZP3/ZP4 complex but do not exist on single components or whether the potential sperm-binding sites on single components become active by complex formation. In this study, we found that recombinant bovine ZP4 adsorbed to plastic wells exhibited sperm-binding activity in the absence of ZP3. We also examined the sperm-binding regions on ZP4 using the solid support assay.

## Materials and methods

### Construction of recombinant baculovirus transfer plasmids for bovine ZP proteins

The construction of pBACgus6 transfer plasmids encoding mature polypeptides of ZP2, ZP3, and ZP4, and ZP4 fragment from Asp-136 to Arg-464 (ZP4 (136–464)) with N-terminal S-tag and 6×His-tag (His-tag) was reported previously [20]. The construction of pBACgus6 transfer plasmid encoding N-terminally S-tagged along with FLAG-tagged mature ZP4 polypeptide was also reported previously [20]. The preparation of pBACgus6 plasmids encoding N- or C-terminal deletion mutants of ZP4 was performed with the PrimeSTAR Mutagenesis Basal Kit (Takara, Kyoto, Japan) using the baculovirus transfer plasmid for His- and S-tagged ZP4 as a template (see Fig 1). The preparation of pBACgus6 plasmids encoding enhanced green fluorescent protein (EGFP) was performed as follows. The cDNA fragment encoding EGFP was amplified using pEGFP-N1 (Clontech, Mountain View, CA, USA) as a template and using sense primer with Sac II site and antisense primer with Nhe I site. The amplified fragments were digested with Sac II and Nhe I and inserted into the corresponding sites of pBACgus6 encoding mature ZP4. Then the plasmid was subjected to deletional mutagenesis with the PrimeSTAR Mutagenesis Basal Kit and the region coding from Lys-25, N-terminus of mature ZP4, to Ala-289 and the region coding from Leu-341 to Arg-464, C-terminus of mature ZP4, were deleted successively to obtain pBACgus6 coding His-tagged EGFP fused to ZP4 fragment from Ser-290 to Lys-340 (ZP4(290–340)). In order to obtain pBACgus6 coding His-tagged EGFP, the region coding mature ZP4 was deleted from pBACgus6 coding His-tagged EGFP fused to mature ZP4 with the PrimeSTAR Mutagenesis Basal Kit. The DNA sequences of the constructed plasmids were confirmed using a commercial DNA sequencing service (Macrogen, Fukuoka, Japan).

### Recombinant baculovirus

The procedure for the preparation of recombinant viruses for His- and S-tagged ZP2, ZP3, and ZP4 and FLAG- and S-tagged ZP4 was previously reported [20]. In this study, we prepared new recombinant baculoviruses as follows. Sf9 cells were routinely propagated in Sf-900II serum-free medium (Invitrogen, Carlsbad, CA, USA). Transfer plasmid DNA preparations containing individual complementary DNAs of the ZP4 mutants were transfected along with flashBAC DNA (Oxford Expression Technologies, Oxford, UK) into Sf9 cells according to the manufacturer’s protocol.

All recombinant ZP proteins were expressed as secretory proteins using signal peptide derived from pBACgus6. The expression and secretion of each recombinant protein into the culture supernatant were examined as previously reported [20].

### Purification of recombinant ZP proteins from culture supernatants

For large-scale protein production, Sf9 cells (200 mL at 1.0 × 10^6^ cells/mL) were infected with a corresponding recombinant virus or a mixture of corresponding recombinant viruses. After 48 h of culture in suspension, the medium was centrifuged at 800 × g for 10 min, and the supernatant fraction was filtered through a 0.45 μm filter. The filtered supernatants were subjected to metal-chelation column chromatography using TALON resin (Clontech) equilibrated with wash buffer (20 mM Tris-HCl, pH 7.9, and 0.15 M NaCl) at a flow rate of 0.5 mL/min at 4°C. The column was washed with 10-column volumes of the wash buffer, and the bound protein was eluted with 6-column volumes of the wash buffer containing 150 mM imidazole. Protein concentrations were determined with absorbances at 280 nm considering absorbances at 1 mg/mL which are calculated from amino acid compositions of each protein (https://web.expasy.org/protparam/). Protein yield was typically 20 μg from a 200-mL culture.

For the purification of mixtures including ZP4 (ZP2/ZP4, ZP3/ZP4, and ZP2/ZP3/ZP4), Sf9 cells were infected with a mixture of recombinant viruses encoding His-tagged ZP2, His-tagged ZP3, and FLAG-tagged ZP4 (instead of His-tagged ZP4) in the corresponding combination, and the culture supernatants were subjected to TALON column chromatography as described above.

### SDS-PAGE

SDS-PAGE was performed on a 12.5% (w/v) separating gel under reducing conditions according to the Laemmli method [24]. Standard proteins with a broad molecular mass range (Takara) were used to estimate protein molecular masses and the gels were silver-stained. For westernblots, prestained molecular mass marker (SMOBIO Tech, Taiwan) was used.

### Biotinylation of recombinant ZP proteins

Recombinant ZP proteins purified with TALON column chromatography were dialyzed against phosphate-buffered saline (PBS). Biotinamidohexanoic acid 3-sulfo-N-hydroxysuccinimide ester sodium salt (B1022, Sigma-Aldrich) was added to each protein solution to give a molar excess of 40 times over the amount of each protein. Following 2 h incubation at room temperature, the reaction was stopped by adding 1 M Tris-HCl (pH 7.5) to the reaction solution to give a final concentration of 10 mM. Biotinylated ZP proteins were subjected to SDS-PAGE and were transferred to Immobilon-P membrane (Millipore, Bedford, MA, USA). The membrane was blocked with 3% bovine serum albumin (BSA) in Tris-buffered saline (TBS) for 1 h. The membrane was then incubated for 2 h with anti His-tag antibody (Wako, Kyoto, Japan) 3000-fold diluted with TBS containing 1% BSA, washed three times for 15 min each with TBS containing 0.05% Tween-20 (T-TBS) and then incubated for 1.5 h with horseradish peroxidase (HRP)-conjugated rabbit anti-mouse IgG (Wako) 1000-fold diluted with TBS containing 1% BSA. After washing three times for 15 min each with T-TBS, colors were developed using 3,3’,5,5’-tetramethylbenzidine (TMB) (SeraCare, Gaithersburg, MD, USA). The same membrane was stripped in Stripping solution (Wako) and blocked with 3% BSA in TBS. The membrane was reprobed for 2 h with 0.5 μg/mL HRP-conjugated streptavidin (Sigma-Aldrich) in TBS containing 1% BSA, washed three times for 15 min each with T-TBS, and colors were developed using TMB.

### Adsorption of ZP proteins to plastic wells and quantitation of adsorbed ZP proteins

Recombinant ZP proteins were adsorbed to each well of a 96-well plate (Nalge Nunc, Rochester, NY, USA) overnight at 4°C. After a rinse with PBS, the wells were blocked with 3% BSA in TBS for 1 h at room temperature. The wells were washed three times with TBS. An antibody against His-tag or FLAG-tag (Wako) 3,000-fold diluted with TBS containing 1% BSA was added to the wells according to the tag of the recombinant ZP protein and allowed to incubate for 1 h at room temperature. After washing three times with T-TBS, the wells were incubated with HRP-conjugated anti-mouse IgG antibody (Wako) 1,000-fold diluted with TBS containing 1% BSA for 1 h at room temperature. After washing three times with T-TBS, 2,2’-azinobis (3-ethylbenzothiazolin-6-sulfonic acid) (Roche) was added to the wells as a substrate of HRP. After incubation for 1 h at room temperature, the absorbance at 405 nm was measured with a plate reader (TECAN, Austria).

### Sperm binding to ZP proteins adsorbed to plastic wells (solid support assay)

**Fig 2:**
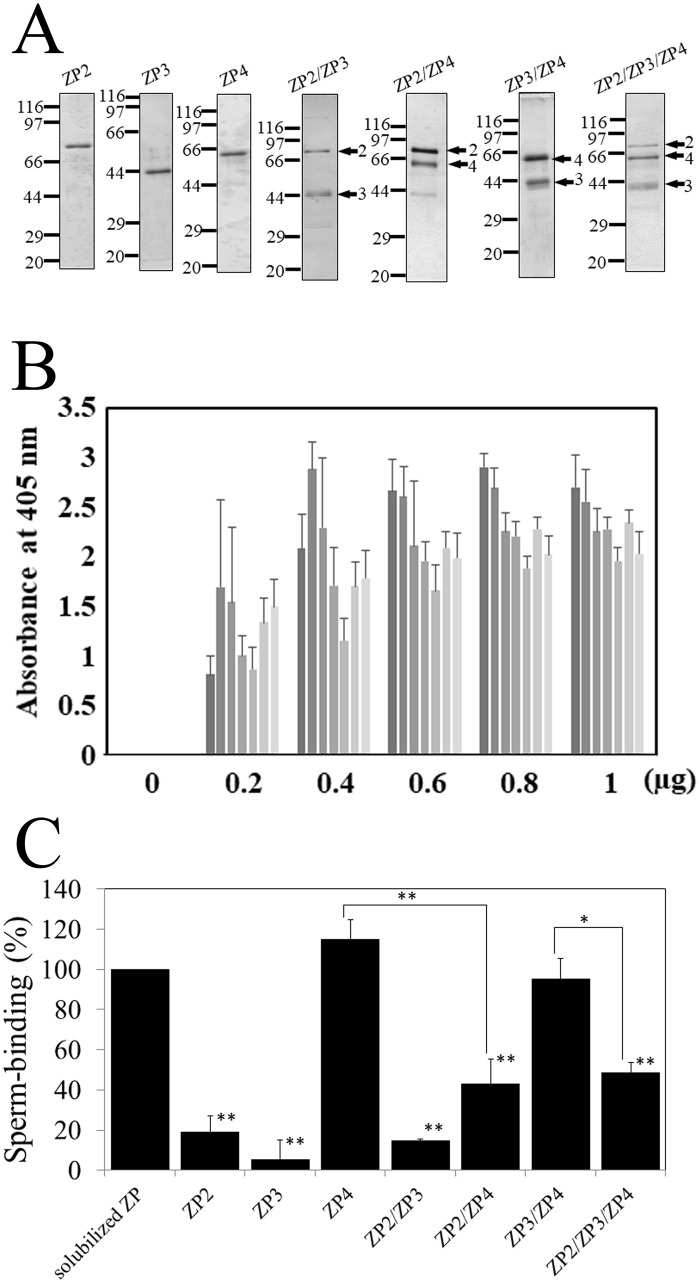
Sperm-binding activity of bovine ZP proteins in a solid supported form. (A) Sodium dodecyl sulfate polyacrylamide gel electrophoresis (SDS-PAGE) of recombinant bovine ZP proteins. Each single component of bovine ZP, ZP2, ZP3, and ZP4; combinations of two components, ZP2/ZP3, ZP2/ZP4, and ZP3/ZP4; and combinations of all three components, ZP2/ZP3/ZP4; were expressed in Sf9 cells by infecting the corresponding baculoviruses and partially purified using TALON resin specific to the His-tag. SDS-PAGE of elution fractions are shown. Gels were silver-stained. Molecular mass standards (kDa) are indicated on the left side of each panel. In the gels loaded with two or three component mixtures, each band is shown by an arrow with a number (ZP2:2, ZP3:3, and ZP4:4). (B) Adsorption of recombinant bovine ZP proteins to plastic wells. The amount of each recombinant ZP protein or mixture added to one plastic well is indicated under each group of bars. The amount of proteins necessary for adsorption saturation was examined by detecting the adsorbed proteins with antibodies specific to N-terminal His-tag and N-terminal FLAG-tag. The experiment was performed three times and average±standard deviation (SD) of absorbance at 405 nm is shown. The amounts of ZP2, ZP3, ZP4, ZP2/ZP3, ZP2/ZP4, ZP3/ZP4, and ZP2/ZP3/ZP4 adsorbed to plastic wells are shown by bars with the gradation from dark gray (left) to light gray (right), respectively, in each group of seven bars. (C) Sperm-binding activity of bovine ZP proteins. Plastic wells were coated with the ZP proteins indicated in the graph. The number of sperm bound to wells coated with solubilized native ZP varied from 28 to 72 among experiments but was designated as 100% at each experiment. Assays were repeated at least three times. Data are presented as the mean ± SD, with statistical significance relative to “solubilized ZP” indicated as P < 0.01 (**) on the right side of the SD bars. The statistical significance between ZP4 and ZP2/ZP4, and between ZP3/ZP4 and ZP2/ZP3/ZP4, is indicated as P < 0.01 (**) and P < 0.05 (*) above the respective lines.

Recombinant bovine ZP protein (0.8 or 1.0 μg as described in the text or figure legends in 50 μL of wash buffer containing 150 mM imidazole) or solubilized bovine ZP (positive control in Fig 2, 0.3 μg in 50 μL of PBS) was added to each well of a 96-well plate (Nalge Nunc) and incubated at 4°C overnight. The wells were rinsed with PBS and blocked with TBS containing 3% BSA at 38.5°C for 2 h. We used frozen bovine sperm from Holstein bulls. The sperm straws supplied for artificial insemination were purchased from Animal Genetics Japan Co., Ltd. (Mie, Japan). Frozen bovine sperm was thawed and washed twice in pre-warmed (38.5°C) Brackett and Oliphant (BO) solution without BSA [20, 23, 25]. The bovine sperm was then capacitated by incubation in BO solution containing BSA for 30 min. Capacitation and subsequent incubations were carried out at 38.5°C under 2% CO_2_. The wells of the plate were rinsed three times with PBS, 50 μL aliquots containing 4 × 10^5^ capacitated sperm were transferred into the wells, and the plates were incubated for 2 h. The wells were washed three times with BO solution, 50 μL of 70% glycerol in PBS was added to each well, and the sperm bound to the wells was recovered by 20 strokes of vigorous pipetting. The number of sperm in 0.1 μL of suspension was determined using a haemocytometer. The number of sperm bound to the wells not coated with recombinant proteins or solubilized ZP (0 to 5) was subtracted from the number of sperm bound to the wells coated with recombinant proteins or solubilized ZP. The average number of sperm in positive control experiments (100% sperm binding) is shown in the legends for Figs 2, 4–6.

### Competitive inhibition assay

Competitive inhibition assay was performed in 96-well plates, as described previously [20]. Briefly, recombinant bovine ZP2/ZP3/ZP4 mixture (1.0 μg in 50 μL of TALON wash buffer containing 150 mM imidazole) was adsorbed to each well of a 96-well plate (Nalge Nunc). The wells were blocked with BSA. Frozen bovine sperm were thawed, washed, and then capacitated as describe above. Each of biotinylated recombinant ZP2 (0.38 μg), ZP3 (0.32 μg) and ZP4 (0.37 μg) was preincubated at room temperature for 30 min in the presence or absence of 0.03 μg of streptavidin (Sigma-Aldrich) in 50 μL solution and then mixed with 50 μL of sperm suspension containing 4 × 10^5^ sperm. Each mixture was incubated at 38.5°C for 30 min and then transferred into the wells, and the plates were incubated at 38.5°C for 2 h. The wells were then washed three times with BO solution, 50 μL of 70% glycerol in PBS was added to each well; sperm bound to the wells were recovered by 20 strokes of vigorous pipetting. The number of sperm in 0.1 μL of suspension was determined using a haemocytometer. The number of sperm bound to the wells not coated with recombinant bovine ZP2/ZP3/ZP4 protein mixture (0 to 5) was subtracted from the number of sperm bound to the wells coated with recombinant bovine ZP2/ZP3/ZP4 protein mixture. The number of bound sperm in the control experiments without inhibitors (100% sperm binding) is shown in the legend for Fig 2.

### Indirect immunofluorescent staining of recombinant ZP4 bound to sperm

Frozen bovine sperm were washed and capacitated as described above, and each of 50 μL aliquots containing 4 × 10^5^ sperm was incubated with 0.37 μg of biotinylated recombinant ZP4 preincubated in the presence or absence of 0.03 μg of streptavidin at 38.5°C for 30 min. The sperm were washed with PBS three times by centrifugation at 2,000 × *g* for 1 min, suspended in PBS, transferred onto cover glasses, and fixed with 3.7% formaldehyde in PBS at 38.5°C for 30 min. The cover glasses were then rinsed with PBS and blocked with 3% BSA in TBS at 38.5°C for 1 h. The proteins that bound to sperm were detected using anti-porcine ZP4 antibody [20] 100-fold diluted with 1% BSA in TBS as the primary antibody and fluorescein-conjugated goat anti-rabbit IgG antibody (Wako) diluted to 1 μg/mL with 1% BSA in TBS as the secondary antibody. The sperm were observed under a fluorescence microscope (BH20, Olympus).

### Statistical analysis

Welch’s t-test was used to determine whether the numbers of bound sperm in the solid support assay and in the competitive inhibition assay differed significantly between two groups. Differences were considered to be significant at P < 0.05.

## Results

### Bovine ZP4 binds to sperm in the absence of ZP3 in a solid supported form

Bovine ZP2, ZP3, and ZP4 and the combinations of ZP2/ZP3, ZP2/ZP4, ZP3/ZP4, and ZP2/ZP3/ZP4 were expressed using a baculovirus-Sf9 cell system and purified with the use of an N-terminal His-tag (Fig 2A) [20]. The recombinant bovine ZP2, ZP3, and ZP4 correspond to the mature polypeptides Ile-36 to Arg-637, Arg-32 to Arg-348, and Lys-25 to Arg-464, respectively, with N-terminal tags [20]. In the previous study, among the single components or the combination of two or three components, only ZP3/ZP4 and ZP2/ZP3/ZP4 mixtures inhibit the binding of sperm to the plastic wells coated with solubilized ZP in a competitive inhibition assay [20]. In the present study, we examined the sperm-binding activity of these recombinant proteins in a solid supported form by adsorbing the proteins to plastic wells. We first examined amounts of ZP2, ZP3 and ZP4 enough to saturate the adsorption of the proteins to the plastic wells and found that addition of 0.8 μg of protein was enough for saturation of these three proteins (Fig 2B). These three proteins gave similar absorbances at saturation, suggesting that similar number of molecules of each protein were adsorbed to the plastic wells. The number of sperm bound to the wells coated with ZP4 was comparable to that of solubilized ZP, a positive control, whereas the number of sperm bound to the wells coated with ZP2 or ZP3 was much lower than that of solubilized ZP (Fig 2C). Thus, among the three ZP components, ZP4 plays a main role in sperm binding. To see possible cooperation between components on sperm binding, we next examined sperm-binding activities of mixtures of two or three components co-expressed in Sf9 cells. In the previous study for a competitive inhibition of sperm–ZP binding, all three recombinant ZP proteins were N-terminally His-tagged and therefore the heterocomplex fraction obtained by metal-chelate affinity chromatography was actually a mixture of the heterocomplex and each of the individual components [20]. In this study, N-terminally FLAG-tagged ZP4 was expressed instead of His-tagged ZP4 to exclude free ZP4 during the purification when the heterocomplexes including ZP4 were prepared. We examined amounts of ZP2/ZP3, ZP2/ZP4, ZP3/ZP4 and ZP2/ZP3/ZP4 enough to saturate the adsorption of the protein mixtures to the plastic wells and found that addition of 0.8 μg of protein mixture was enough for saturation of these protein mixtures (Fig 2B). The number of sperm bound to the wells coated with the ZP2/ZP3 mixture was at a similar level to those of ZP2 and ZP3 (Fig 2C). The number of sperm bound to the wells coated with the ZP2/ZP4 mixture was higher than that of ZP2/ZP3, but significantly lower than that of ZP4 alone. The number of sperm bound to the wells coated with ZP3/ZP4 was similar to that of ZP4, while the number of sperm bound to the wells coated with ZP2/ZP3/ZP4 was significantly lower than that of ZP3/ZP4. Thus, among the combinations of the three components, the mixtures containing ZP4 showed higher sperm-binding activity than ZP2/ZP3. It is possible that the lower sperm-binding activity of ZP2/ZP4 and ZP2/ZP3/ZP4 reflects lower density of ZP4 on the wells, since we did not add the protein mixtures containing equivalent amount of ZP4 to the wells but adjusted the total amount of recombinant proteins to saturation level. These results suggest that ZP4 adsorbed to the plastic wells binds to sperm with high avidity in a multivalent state, whereas soluble ZP4 does not bind to sperm with an affinity high enough to inhibit sperm–ZP binding in a competitive inhibition assay [20].

### Bovine ZP4 multimerized through biotin binds to sperm acrosomal region in the absence of ZP3

**Fig 3:**
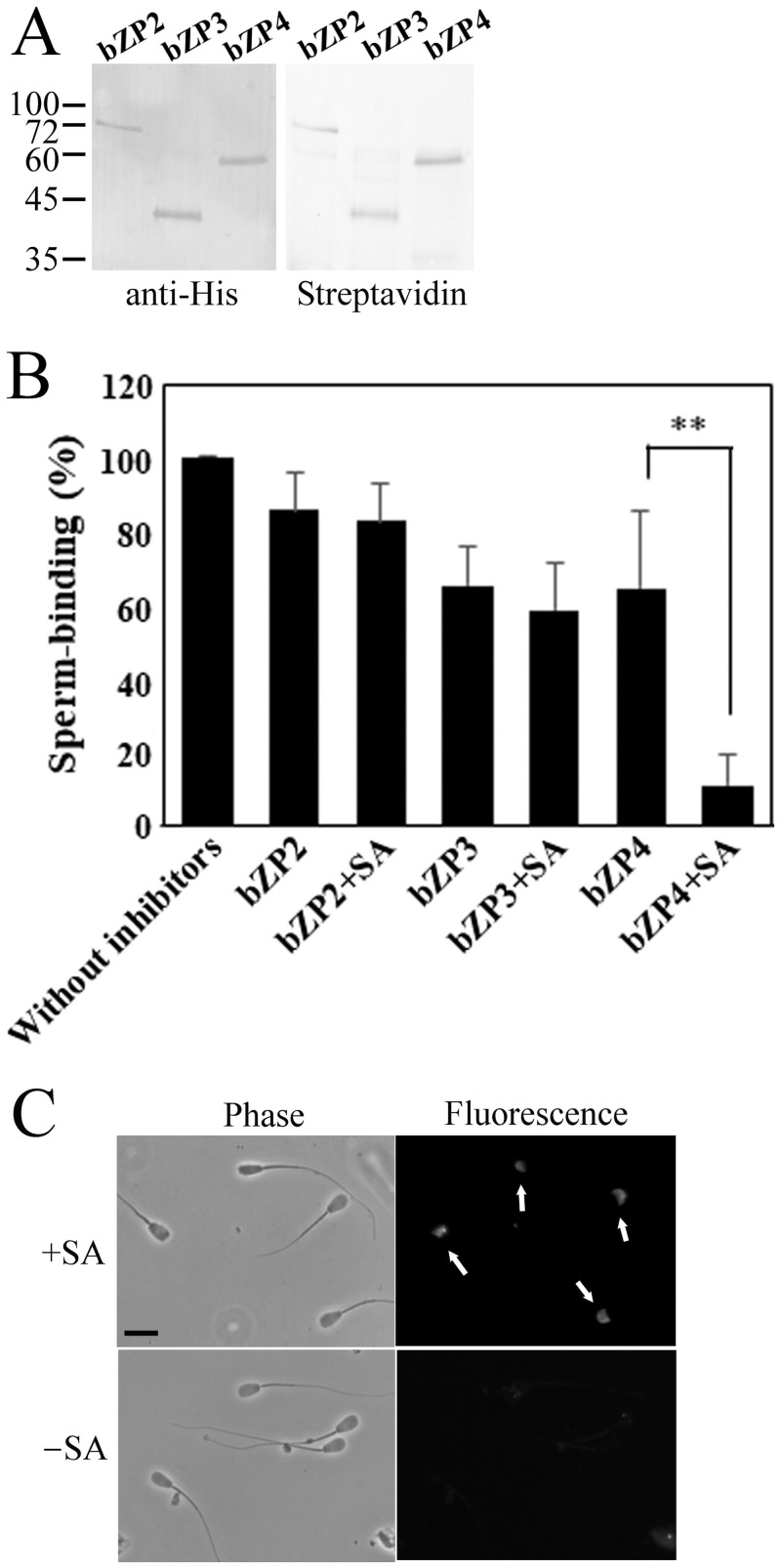
Sperm-binding activity of bovine ZP proteins multimerized in a solution using streptavidin-biotin binding. (A) Biotinylation of bovine ZP proteins. Bovine ZP2, ZP3, and ZP4 were biotinylated as described in Materials and methods section. These proteins were subjected to westernblot and detected with anti-His tag antibody (anti-His) used as a primary antibody (left panel). The same membrane was stripped and reprobed with horseradish peroxidase-conjugated streptavidin (Streptavidin, right panel). Molecular mass markers are indicated on the left of the membrane in kDa. (B) Effect of streptavidin on the competitive inhibition of biotinylated ZP proteins on sperm–ZP binding. Plastic wells were coated with recombinant bovine ZP2/ZP3/ZP4 mixture. Bovine sperm were preincubated with each inhibitor indicated below each bar and then added to the coated wells. bZP2, biotinylated ZP2; bZP3, biotinylated ZP3; bZP4, biotinylated ZP4; SA, streptavidin. The count of sperm bound to the bovine ZP2/ZP3/ZP4-coated wells in the absence of inhibitors (Without inhibitors) varied from 39 to 57 among experiments but was designated as 100% at each experiment. Assays were repeated four times. Data are presented as the mean ± SD. Neither bZP2 nor bZP3 showed significant increase in inhibitory activity by preincubation with SA, while bZP4 showed significant increase in inhibitory activity by preincubation with SA, as indicated by P < 0.01 (**) above the line. (C) Indirect immunofluorescent detection of ZP4 bound to bovine sperm. Biotinylated bovine ZP4 was preincubated with streptavidin (+SA, upper panels) or without streptavidin (–SA, lower panels) and then mixed with bovine sperm. After washings, ZP4 bound to sperm was detected with anti-porcine ZP4 antiserum as a primary antibody and fluorescein isothiocyanate-conjugated anti-rabbit IgG antibody as a secondary antibody. Arrows show that acrosomal region is fluorescently stained. Phase, phase contrast image; Fluorescence, fluorescence image; SA, streptavidin. Bar in the upper left panel indicates 10 μm.

Recombinant bovine ZP3/ZP4 heterocomplex binds to sperm acrosomal region while ZP4 alone does not [20]. Since our present study suggested that ZP4 alone shows sperm-binding activity in a multivalent form, we prepared biotinylated ZP proteins and investigated if the ZP proteins multimerized in a solution using biotin-streptavidin binding show sperm-binding activity. ZP2, ZP3 and ZP4 were biotinylated as revealed by detection with HRP-conjugated streptavidin (Fig 3A). Inhibitory activity of the biotinylated ZP2, ZP3 and ZP4 for sperm–ZP binding was examined in the absence or presence of streptavidin (Fig 3B). Biotinylated ZP4 inhibited the binding of sperm to the plastic wells coated with recombinant bovine ZP2/ZP3/ZP4 mixture in the presence of streptavidin but did not inhibit the binding in the absence of streptavidin. On the contrary, biotinylated ZP2 and ZP3 did not significantly inhibit the binding of sperm to the wells coated with recombinant bovine ZP2/ZP3/ZP4 mixture either in the absence or in the presence of streptavidin. Then, we examined binding site of the biotinylated ZP4 on bovine sperm by indirect immunofluorescent detection of ZP4. Biotinylated ZP4 bound to acrosomal region of sperm in the presence of streptavidin but did not bind to sperm in the absence of streptavidin (Fig 3C). These results show that ZP4 multimerized by biotin-streptavidin interaction binds to acrosomal region and inhibits the binding of sperm to the wells coated with bovine ZP2/ZP3/ZP4 mixture.

### Sperm-binding regions on ZP4

Since ZP4 alone showed predominant sperm-binding activity in the solid support assay, we examined the sperm-binding activity of a variety of fragments of ZP4 using this assay. The mature bovine ZP4 polypeptide expressed in Sf9 cells includes residues Lys-25 to Arg-464 (Fig 1) [20, 21] and is designated ZP4 in this study. ZP4 consists of five regions: the N-terminal ZP-N-like domain (Lys-25 to Pro-135, ZP-N1), the trefoil domain (Asp-136 to Tyr-181), the ZP-N domain (Gly-182 to Ala-289, ZP-N2), the hinge region (Ser-290 to Gln-311), and the ZP-C domain (Pro-312 to Arg-464, ZP-C) (Fig 1). We prepared four N-terminal fragments: ZP4 (25–340), which lacks a large part of ZP-C, ZP4 (25–289), which lacks the hinge region and ZP-C, ZP4 (25–184), which lacks the ZP module and therefore consists of ZP-N1 and the trefoil domain, and ZP4 (25–136), which consists of ZP-N1 only (Fig 1).

**Fig 4:**
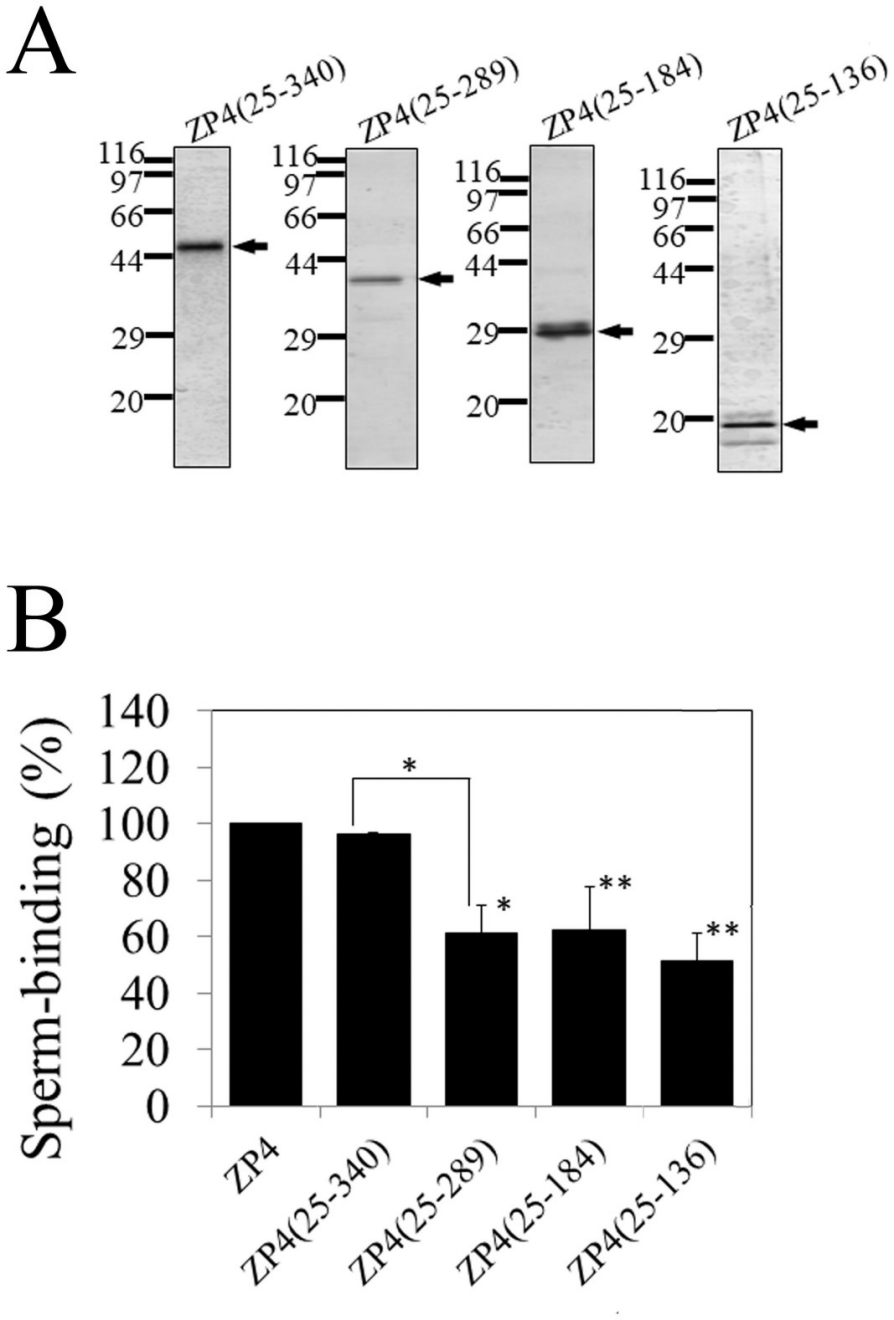
Sperm-binding activity of N-terminal fragments of bovine ZP4. (A) SDS-PAGE of N-terminal fragments of bovine ZP4. Four N-terminal fragments of bovine ZP4, ZP4 (25–340), ZP4 (25–289), ZP4 (25–184), and ZP4 (25–136) were expressed in Sf9 cells and partially purified by TALON resin specific to His-tag. Gels were silver-stained. The objective bands are indicated by arrows. Molecular mass standards (kDa) are indicated on the left side of each panel. (B) Sperm-binding activity of the four N-terminal fragments of bovine ZP4. Plastic wells were coated with the ZP proteins (0.8 μg for each protein) indicated in the graph. The number of sperm bound to wells coated with ZP4 varied from 33 to 65 but was designated as 100% at each experiment. Assays were repeated at least three times. Data are presented as the mean ± SD, with statistical significance relative to ZP4 indicated as P < 0.05 (*) and P < 0.01 (**) on the right side of the SD bars. The statistical significance between ZP4 (25–340) and ZP4 (25–289) is indicated as P < 0.05 (*) above the line. There was no statistical significance among ZP4 (25–289), ZP4 (25–184), and ZP4 (25–136).

These fragments were expressed in Sf9 cells and purified to near homogeneity as revealed by SDS-PAGE (Fig 4A) and adsorbed to the plastic wells. The number of bovine sperm bound to the wells coated with ZP4 (25–340) was not significantly different from that of ZP4 (Fig 4B), which indicates that the C-terminal region from residues 341 to 464 is not necessary for the sperm-binding activity of ZP4. On the other hand, the number of bovine sperm bound to the wells coated with ZP4 (25–289) was significantly lower than that of ZP4 (25–340) (Fig 4B), which indicates that the region between residues 290 and 340 is involved in the sperm-binding activity of ZP4. ZP4 (25–136) showed sperm-binding activity that was not significantly different from that of ZP4 (25–184) or ZP4 (25–289) (Fig 4B), indicating that the region between residues 25 and 136 has sperm-binding activity and that the region between residues 137 and 289 consisting of the trefoil domain and ZP-N2 domain is not necessary for the sperm-binding activity.

**Fig 5:**
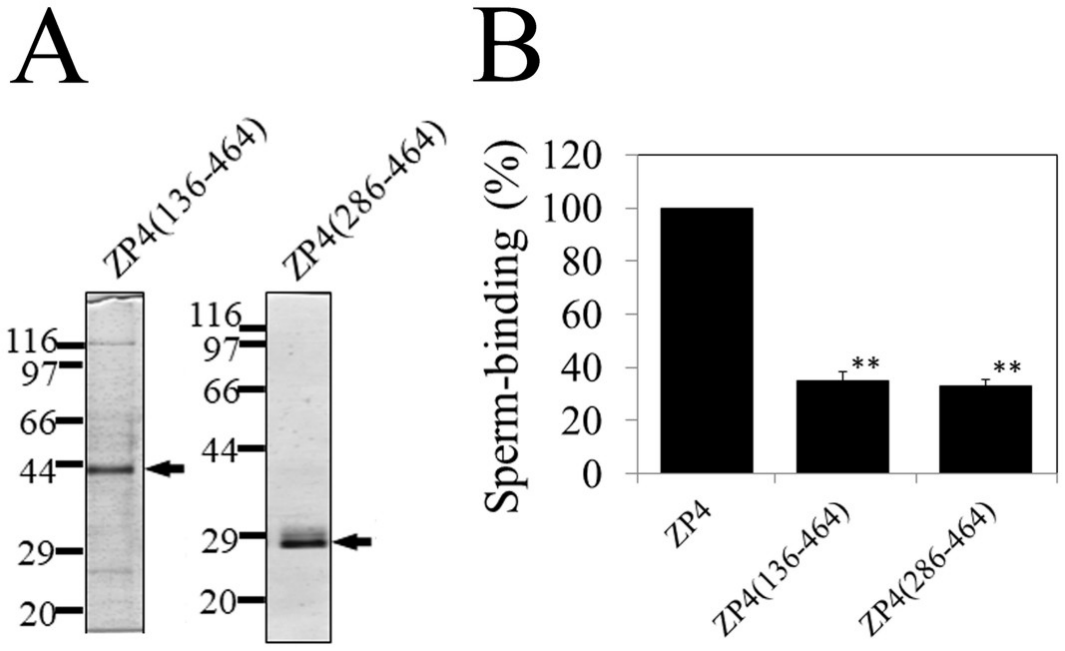
Sperm-binding activity of C-terminal fragments of bovine ZP4. (A) SDS-PAGE of C-terminal fragments of bovine ZP4. Two C-terminal fragments of bovine ZP4, ZP4 (136–464) and ZP4 (286–464) were expressed in Sf9 cells and partially purified by using TALON resin specific to His-tag. Gels were silver-stained. The objective bands are indicated by arrows. Molecular mass standards (kDa) are indicated on the left side of each panel. (B) Sperm-binding activity of the two C-terminal fragments of bovine ZP4. Plastic wells were coated with the ZP4 fragments (0.8 μg for each fragment) indicated in the graph. The number of sperm bound to wells coated with ZP4 varied from 33 to 65 but was designated as 100% at each experiment. Assays were repeated at least three times. Data are presented as the mean ± SD, with statistical significance relative to ZP4 indicated as P < 0.01 (**) on the right side of the SD bars. There was no statistical significance between ZP4 (136–464) and ZP4 (286–464).

To further characterize sperm-binding regions on ZP4, we also prepared two C-terminal fragments: ZP4 (136–464), which lacks ZP-N1, and ZP4 (286–464), which lacks ZP-N1, the trefoil domain, and ZP-N2 (Fig 1). These fragments were expressed in Sf9 cells and purified to near homogeneity as revealed by SDS-PAGE (Fig 5A) and adsorbed to the plastic wells. The numbers of bovine sperm bound to the wells coated with ZP4 (136–464) and ZP4 (286–464) were similar but reduced to approximately 35% of that of ZP4 (Fig 5B). This result indicates that neither the region between residues 137 and 184 corresponding to the trefoil domain nor the region between 185 and 289 corresponding to ZP-N2 is necessary for the sperm-binding activity of ZP4. The reduction in the sperm-binding activity of these C-terminal fragments of ZP4 compared to that of ZP4 can be explained by the loss of the N-terminal sperm-binding region between residues 25 and 136 that roughly corresponds to ZP-N1.

**Fig 6:**
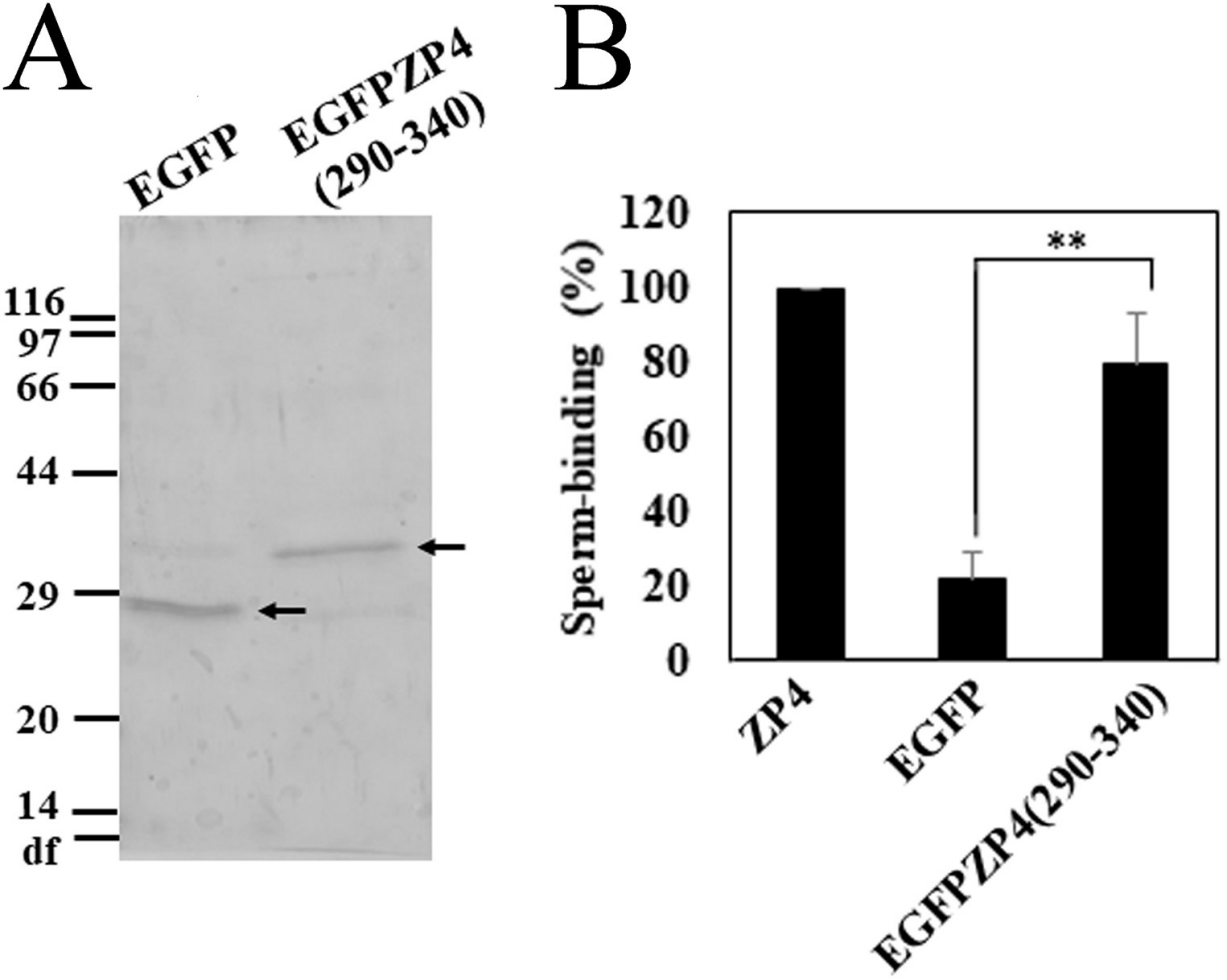
Sperm-binding activity of the region from Ser-290 to Lys-340 of bovine ZP4. (A) SDS-PAGE of enhanced green fluorescent protein (EGFP) and the fragment from Ser-290 to Lys-340 of bovine ZP4 fused to EGFP (EGFPZP4 (290–340)). These proteins were expressed in Sf9 cells and partially purified by using TALON resin specific to His-tag. Gels were silver-stained. The objective bands are indicated by arrows. Molecular mass standards (kDa) are indicated on the left side of the gel. df, dye front. (B) Sperm-binding activity of EGFP and EGFPZP4 (290–340). Plastic wells were coated with the recombinant proteins (1.0 μg for each protein) indicated in the graph. The number of sperm bound to wells coated with ZP4 varied from 32 to 64 among experiments but was designated as 100% at each experiment. Assays were repeated five times. Data are presented as the mean ± SD, with statistical significance between EGFP and EGFPZP4 (290–340) indicated as P < 0.01 (**) above the line.

Since deletion of the region between residues 290 and 340 from ZP4 (25–340) fragment showed significant decrease in the sperm-binding activity of the fragment (Fig 4B), we further examined whether the region between residues 290 and 340 has sperm-binding activity. It is difficult to analyze the ZP4 (290–340) fragment on SDS-PAGE due to the low molecular mass of the fragment. Then we prepared ZP4 (290–340) fragment N-terminally fused to EGFP (EGFPZP4 (290–340)) and EGFP without ZP4 fragment as a control. These proteins were expressed in Sf9 cells and purified to near homogeneity as revealed by SDS-PAGE (Fig 6A) and adsorbed to the plastic wells. The number of bovine sperm bound to the wells coated with EGFPZP4 (290–340) was significantly higher than that of EGFP (Fig 6B), which indicates that the region between residues 290 and 340 has sperm-binding activity. Taken together, these results indicate that the two regions of ZP4 (the region between residues 25 and 136 roughly corresponding to ZP-N1 and the region between residues 290 and 340, including the flexible hinge region and the N-terminal part of ZP-C) have sperm-binding activity.

## Discussion

Previous study has shown that ZP4 in a solution does not inhibit sperm–ZP binding in a competitive inhibition assay and does not bind to sperm as revealed by indirect immunostaining [20]. In the present study, we found that ZP4 shows sperm-binding activity in a solid supported form and in a multimerized form through biotin–streptavidin binding. Taken together these previous and new results, monomeric form of ZP4 may have only a low affinity for sperm, and ZP4 shows high avidity for sperm in a multivalent state. In the previous study, ZP3/ZP4 mixture co-expressed in Sf9 cells inhibited sperm–ZP binding in a competitive inhibition assay and bound to sperm as revealed by indirect immunostaining, while ZP2/ZP4 mixture did not inhibit sperm–ZP binding [20]. In the present study, ZP2/ZP4 mixture showed significantly lower sperm-binding activity than ZP3/ZP4 mixture. Taken together these results, ZP4 may become sperm-binding active in the form of heterocomplex with ZP3 but not in the form of heterocomplex with ZP2.

In the present study, we also found that N-terminal ZP-N1 of ZP4 has higher sperm-binding activity than the region between residues 290 and 340 of ZP4. According to the previous study, however, the N-terminal ZP-N1 of ZP4 is dispensable for the sperm-binding activity of bovine ZP3/ZP4 mixture [20]. The binding of the region between residues 290 and 340 of ZP4 to sperm may be sufficient for inhibition of sperm–ZP binding in a competitive inhibition assay. It is possible that interaction of ZP3 with ZP4 has additional effects on the sperm-binding activity of ZP3/ZP4 complex other than multimerization of ZP4. Recent report has shown that the N-terminal fragment of ZP3 from Arg-32 to Glu-178 interacts with ZP4 and the complex inhibits sperm–ZP binding in a competitive inhibition assay [23]. The mutation of N-glycosylated Asn-146 to Asp in the hinge region of the ZP3 fragment reduces the inhibitory activity of the complex for sperm–ZP binding, while this mutation does not reduce the interaction between the ZP3 fragment and ZP4 [23]. Since ZP3 adsorbed to the plastic wells did not exhibit sperm-binding activity (Fig 2C), the N-glycans at Asn-146 of ZP3 may become sperm-binding active in a form of the ZP3/ZP4 complex. Otherwise, the N-glycosylation at Asn-146 of ZP3 may be necessary for a formation of sperm-binding active site between interfaces of the ZP3 fragment and ZP4. Consistent with this hypothesis, a recent 3D model of pig ZP3/ZP4 complex based on the cryo-EM structure of the filaments of uromodulin pointed out the idea that the hinge region of ZP3 is important for its interaction with ZP4 and for the formation of sperm recognition surface [26].

In pigs, ZP4 without contamination of ZP3 does not have sperm-binding activity but ZP3/ZP4 heterocomplex has the activity [19, 22]. Main sperm-binding sites in the porcine ZP3/ZP4 heterocomplex locate in ZP4 as revealed by using hybrid complexes between native ZP glycoproteins and recombinant ZP glycoproteins [27]. Then, both in cow and pigs, ZP4 plays a major role in sperm recognition. Interestingly, native porcine ZP4 lacks the N-terminal ZP-N1 [28], which could be due to the posttranslational processing between ZP-N1 and the trefoil domain by a processing enzyme, while native bovine ZP4 retains the N-terminal ZP-N1 [9, 21]. This means that the N-terminal ZP-N1 of ZP4 is dispensable for sperm-binding activity of porcine ZP. Identification of sperm proteins which bind to the N-terminal ZP-N1 of bovine ZP4 will be necessary to clarify if this region has a physiological role in sperm–ZP binding in bovine gamete recognition.

Bovine ZP4 contains four potential N-glycosylation sites at Asn-71, -202, -218, and -314. The recombinant ZP4 expressed in Sf9 cells has pauci-mannose type N-glycans with α-Mannose (Man) residues at the nonreducing termini [20]. Asn-71 locates in the N-terminal ZP-N1 of ZP4 and Asn-314 locates in the region between residues 290 and 340. Bovine sperm binds to plastic wells coated with glycolipid analogues and prefers α-Man residues [29]. Nonreducing terminal α-Man residues are essential for the binding of bovine sperm to native ZP [30]. Thus, it is possible that N-glycans of the ZP4 fragments are involved in bovine sperm-binding. This remains to be clarified.

Mouse ZP consists of ZP1, ZP2 and ZP3, while human and rabbit ZP consist of ZP1, ZP2, ZP3 and ZP4. Bovine and porcine ZP lack ZP1 and consist of ZP2, ZP3 and ZP4. *In vivo* and *in vitro* studies showed that N-terminal domain of ZP2 is sperm-binding site on both human and mouse ZP [17, 18], but it is not yet clarified whether the mechanisms in the other species are the same as those in these species or not. Recent report showed rabbit ZP deficient of ZP4 is normal in sperm-binding [31]. But, it is still possible that the sperm-binding activity of ZP4 is a feature unique to bovine and porcine ZP both of which lack ZP1, while it remains to be clarified if ZP4 plays a main role in an *in vivo* fertilization in cow and pigs.

## Supporting information

S1 Fig (ZIP)
