# Supplementary figures and images for: Sperm-binding regions on bovine egg zona pellucida glycoprotein ZP4 studied in a solid supported form on plastic plate
Source: PLoS One. 2021 Jul 9;16(7):e0254234. doi: 10.1371/journal.pone.0254234 (PMC8270413; doi:10.1371/journal.pone.0254234)

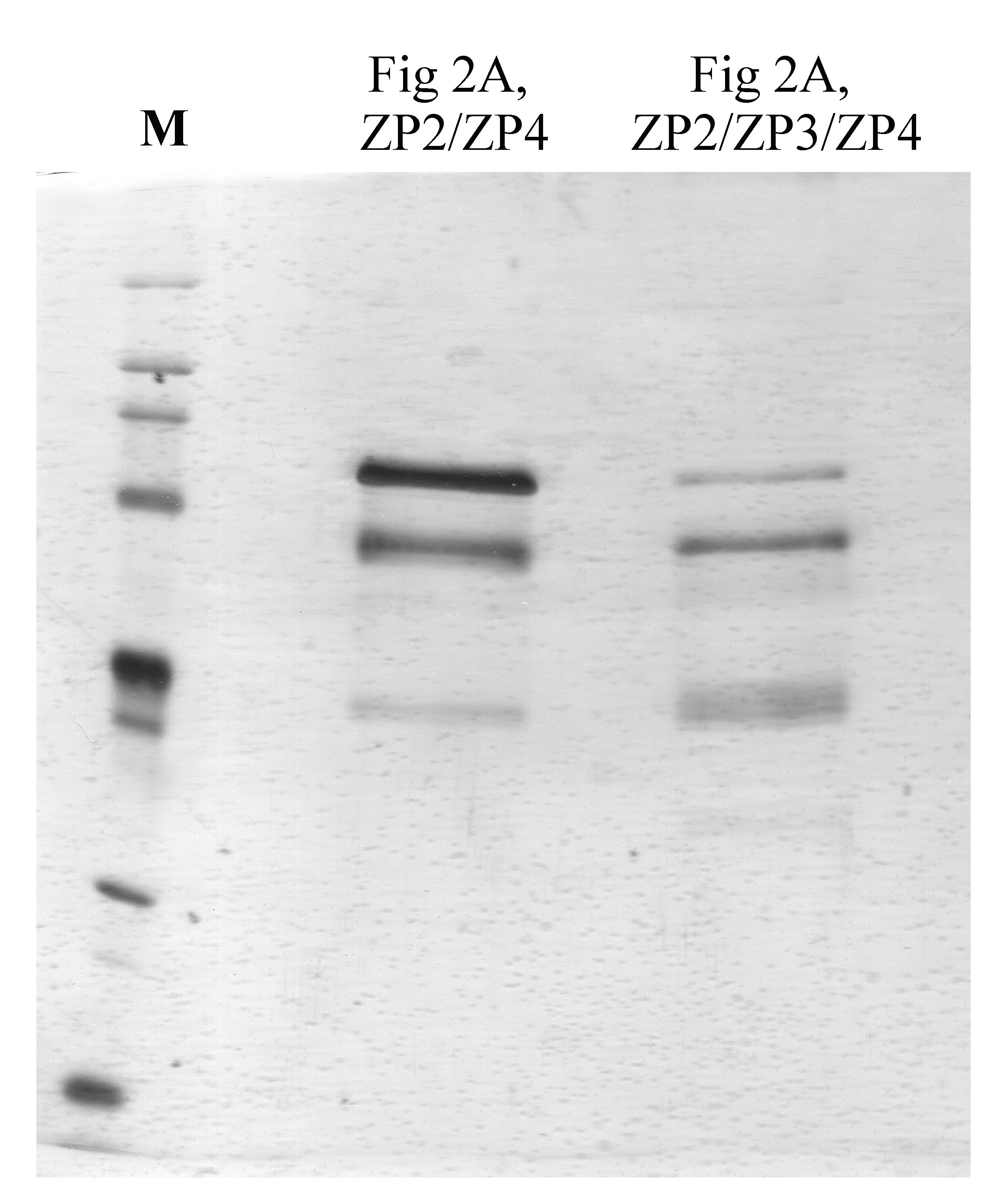

Supplement: S1 Fig — (ZIP) [file pone.0254234.s001.zip › S1 Fig 2A ZP2 and 4 mixture and ZP2 and 3 and 4 mixture.TIF]

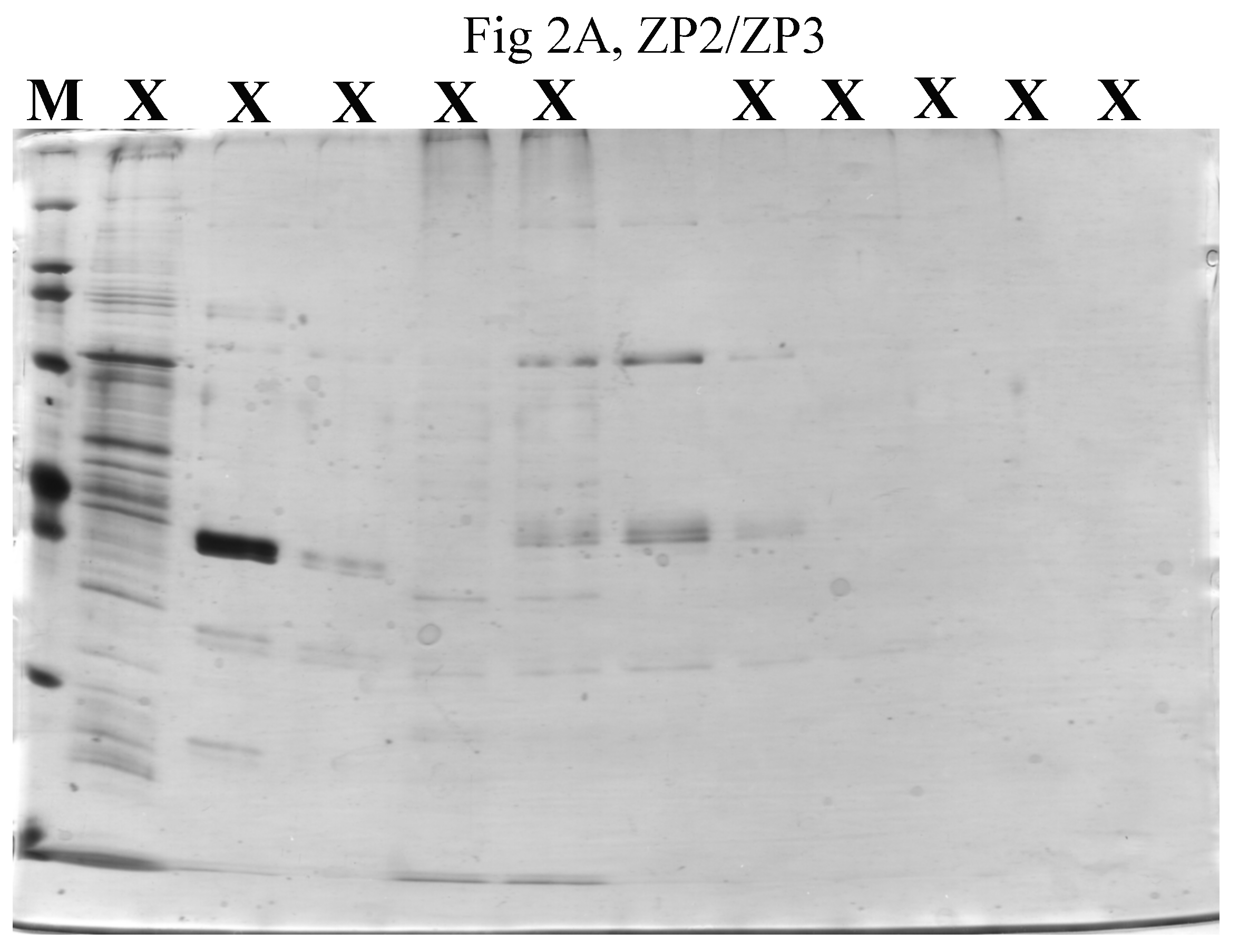

Supplement: S1 Fig — (ZIP) [file pone.0254234.s001.zip › S1 Fig 2A ZP2 and ZP3 mixture.TIF]

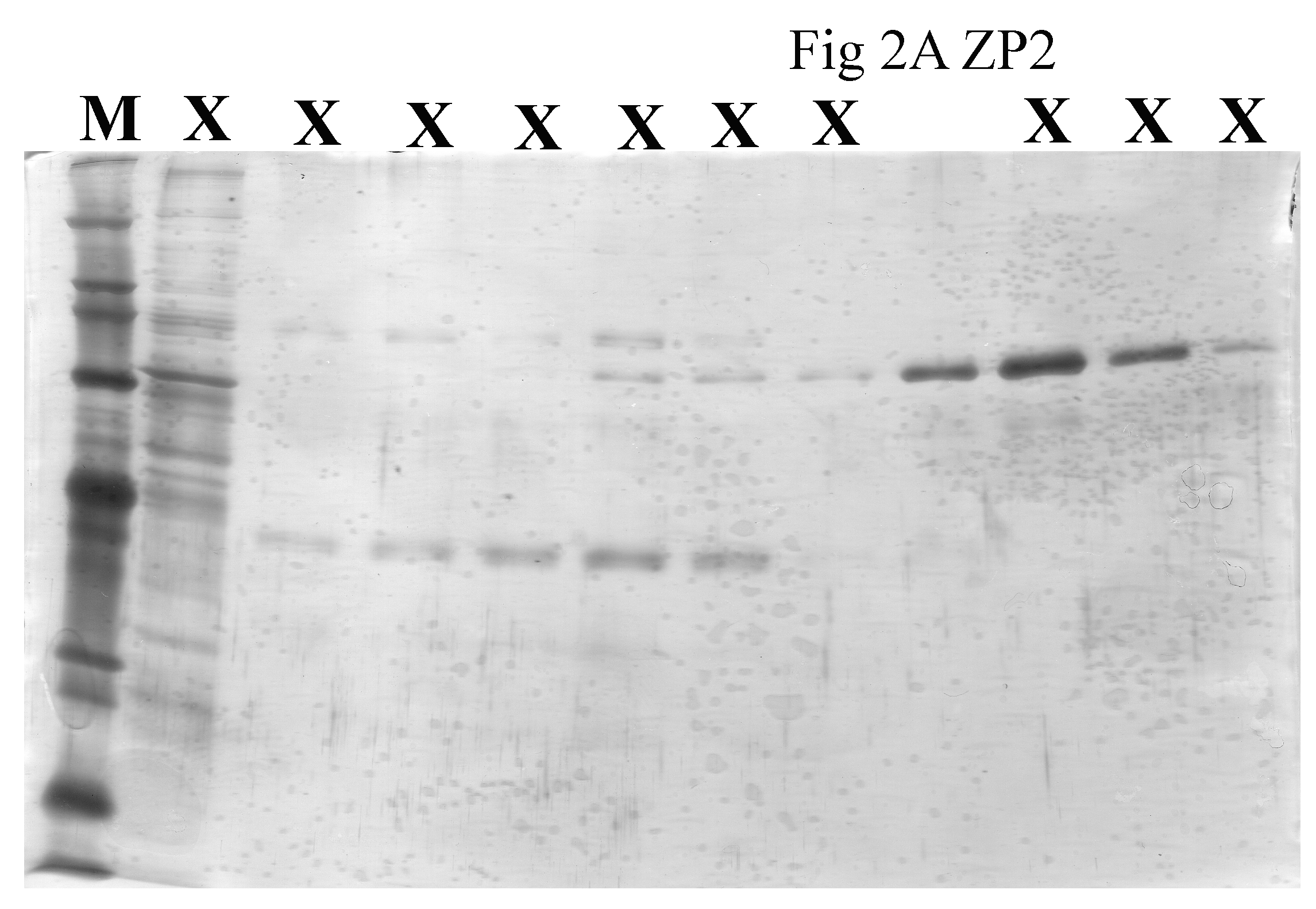

Supplement: S1 Fig — (ZIP) [file pone.0254234.s001.zip › S1 Fig 2A ZP2.TIF]

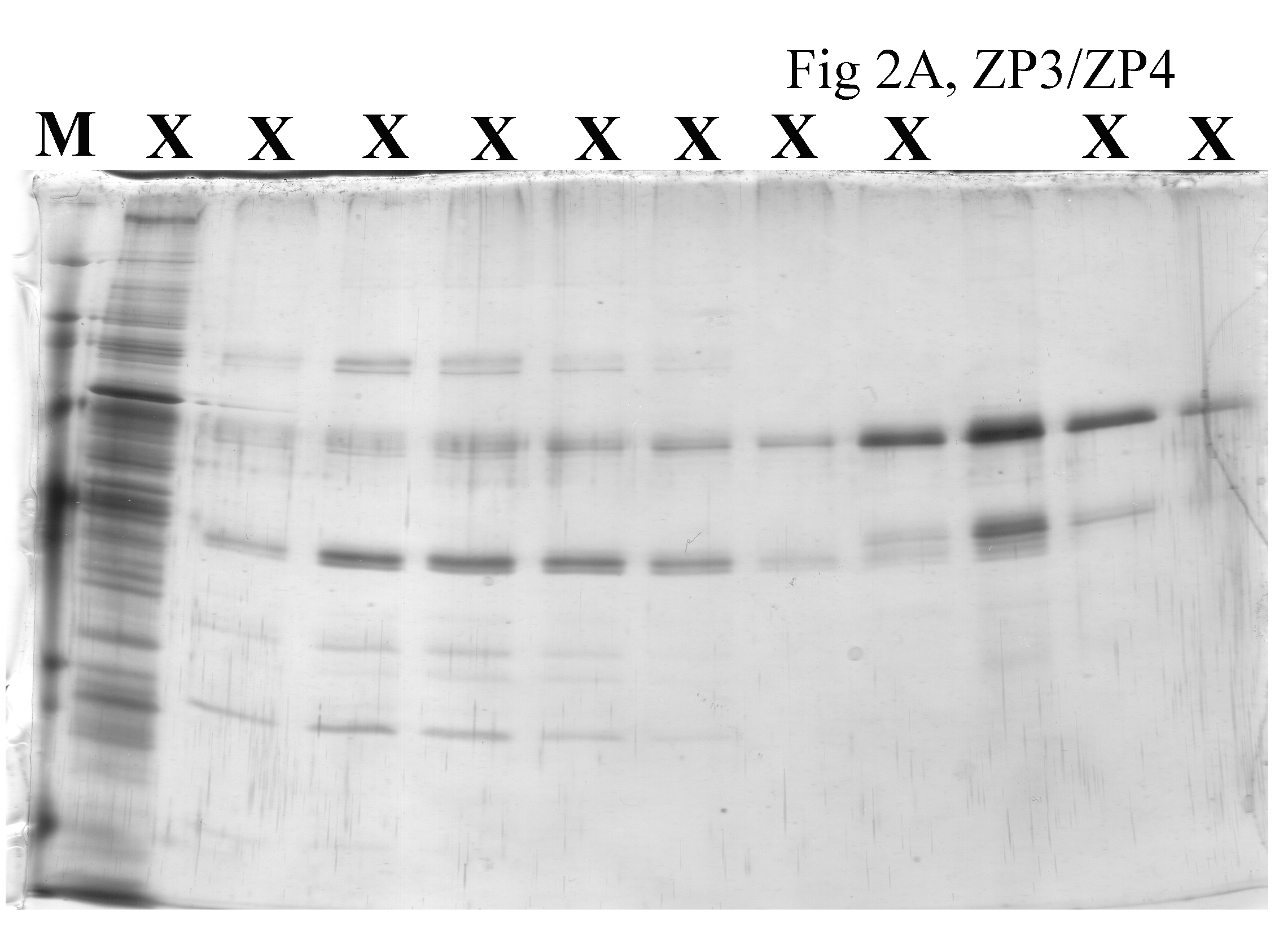

Supplement: S1 Fig — (ZIP) [file pone.0254234.s001.zip › S1 Fig 2A ZP3 and ZP4 mixture.TIF]

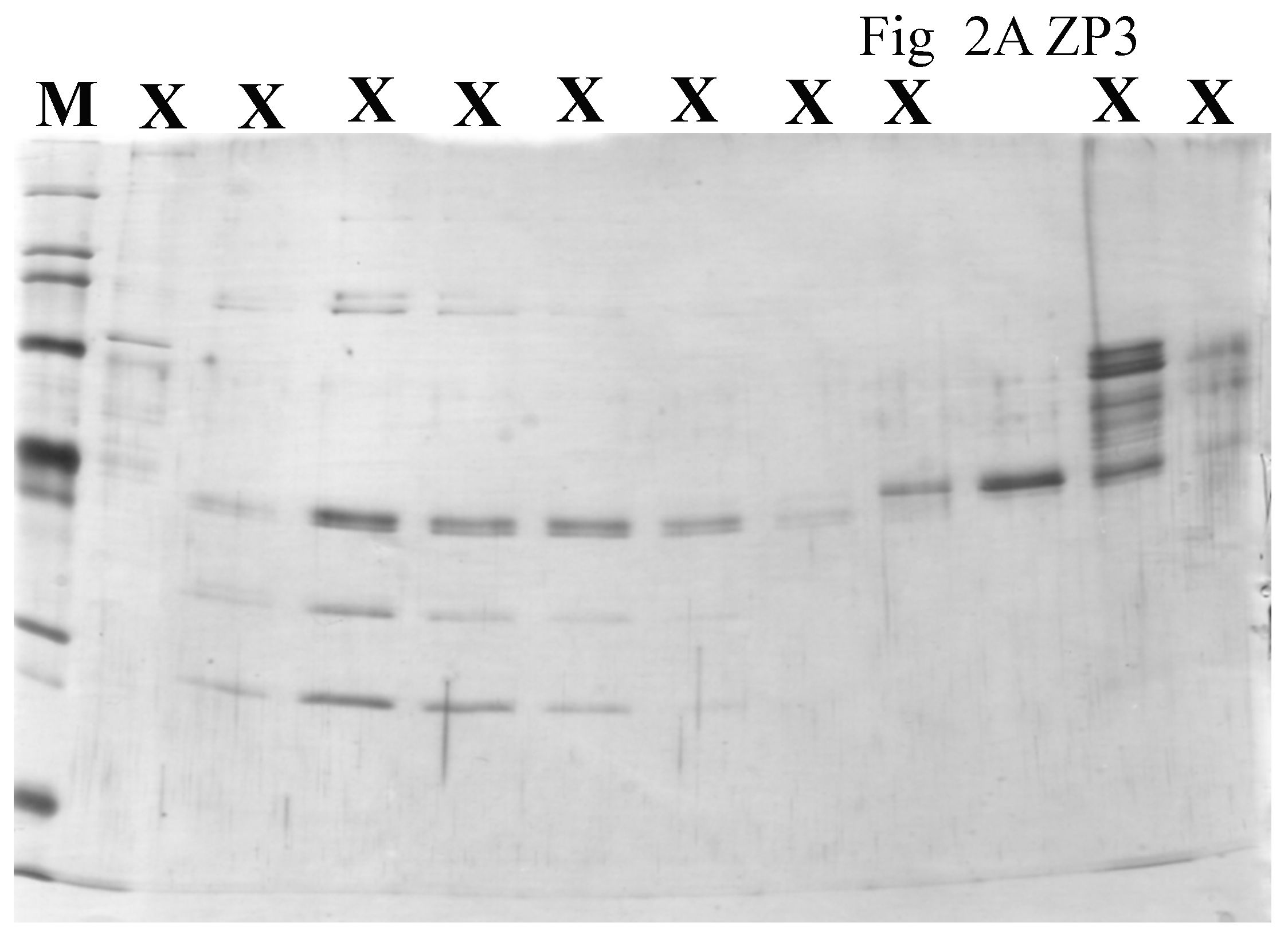

Supplement: S1 Fig — (ZIP) [file pone.0254234.s001.zip › S1 Fig 2A ZP3.TIF]

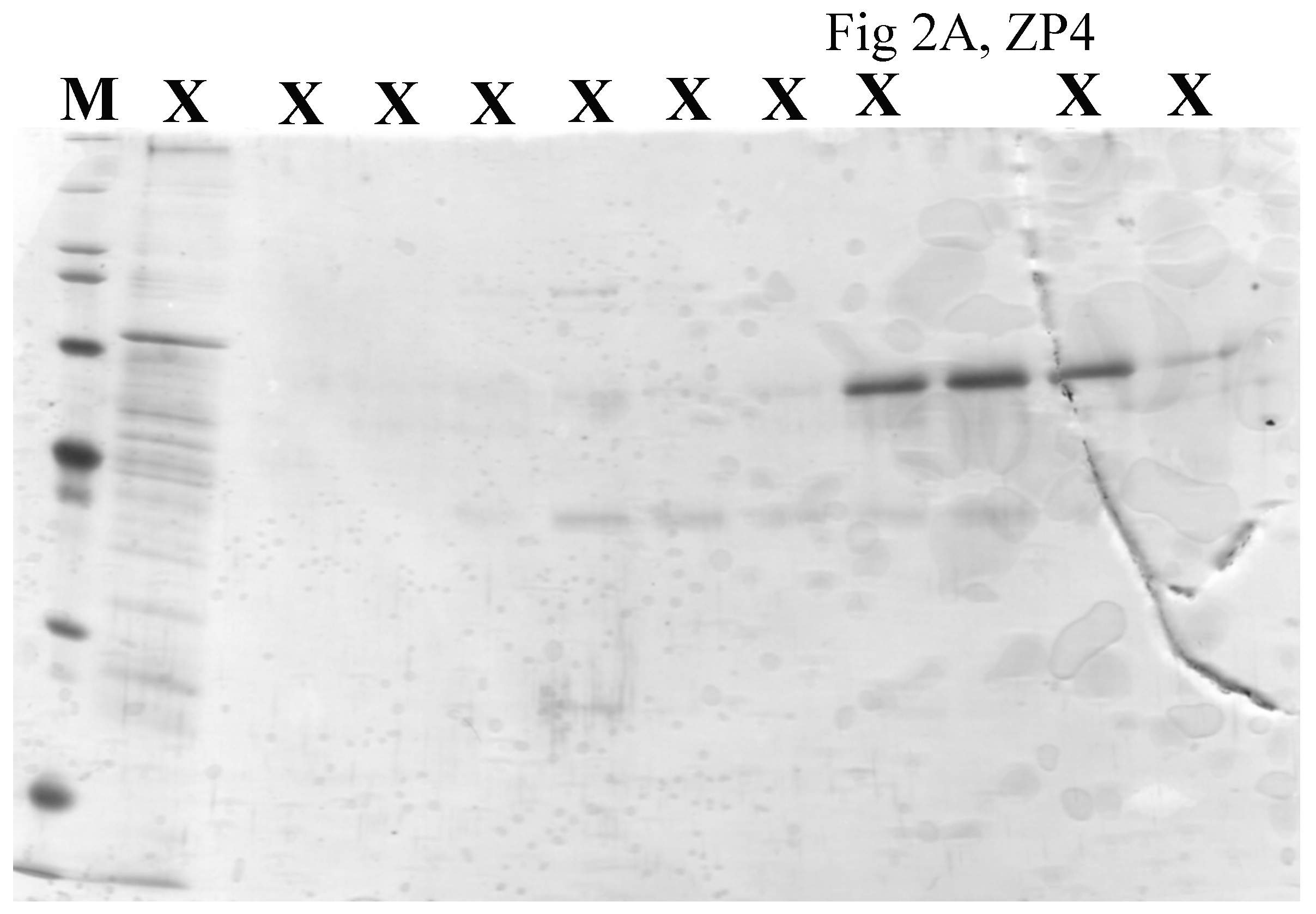

Supplement: S1 Fig — (ZIP) [file pone.0254234.s001.zip › S1 Fig 2A ZP4.TIF]

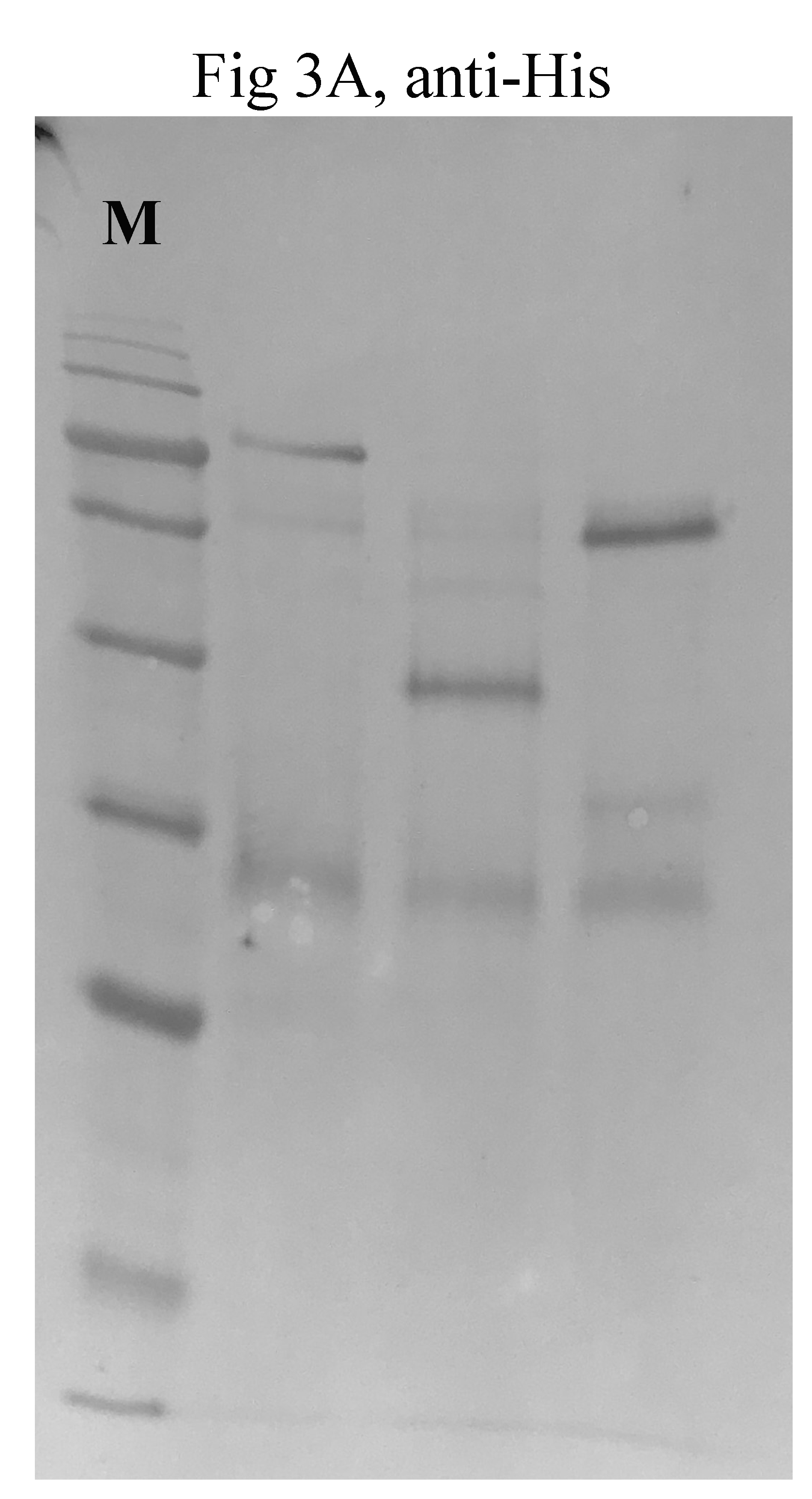

Supplement: S1 Fig — (ZIP) [file pone.0254234.s001.zip › S1 Fig 3A anti-His.TIF]

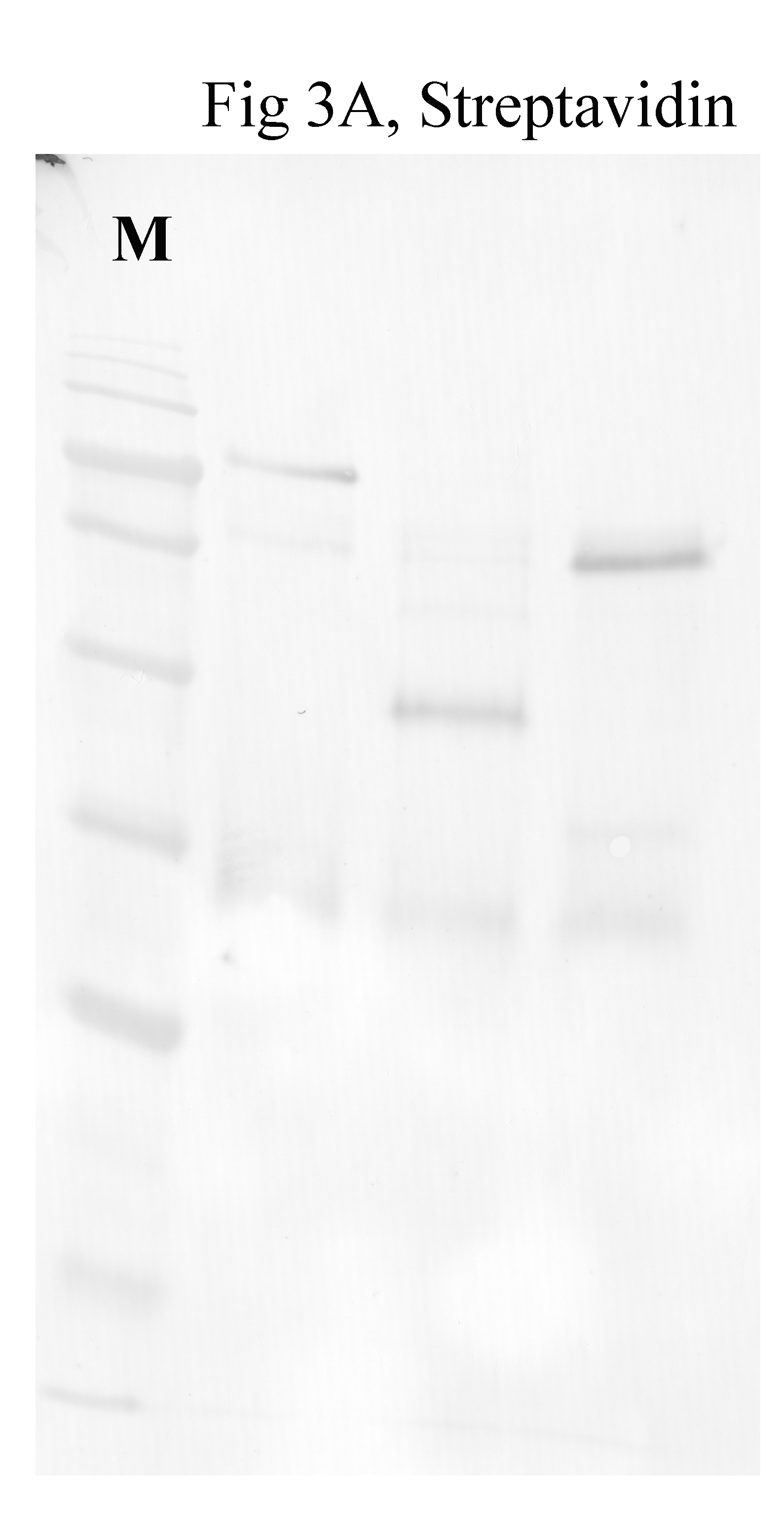

Supplement: S1 Fig — (ZIP) [file pone.0254234.s001.zip › S1 Fig 3A Streptavidin.TIF]

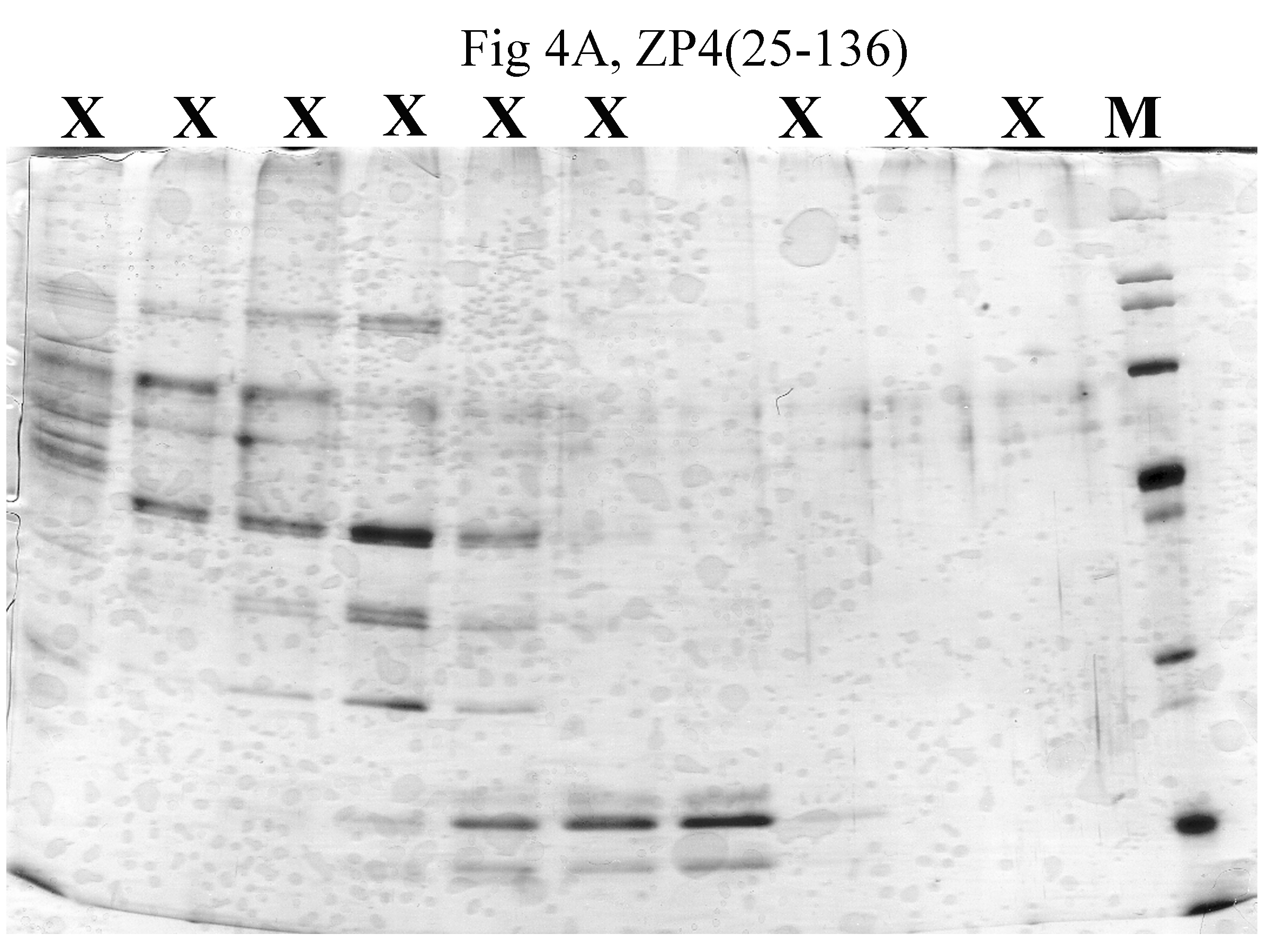

Supplement: S1 Fig — (ZIP) [file pone.0254234.s001.zip › S1 Fig 4A ZP4(25-136).TIF]

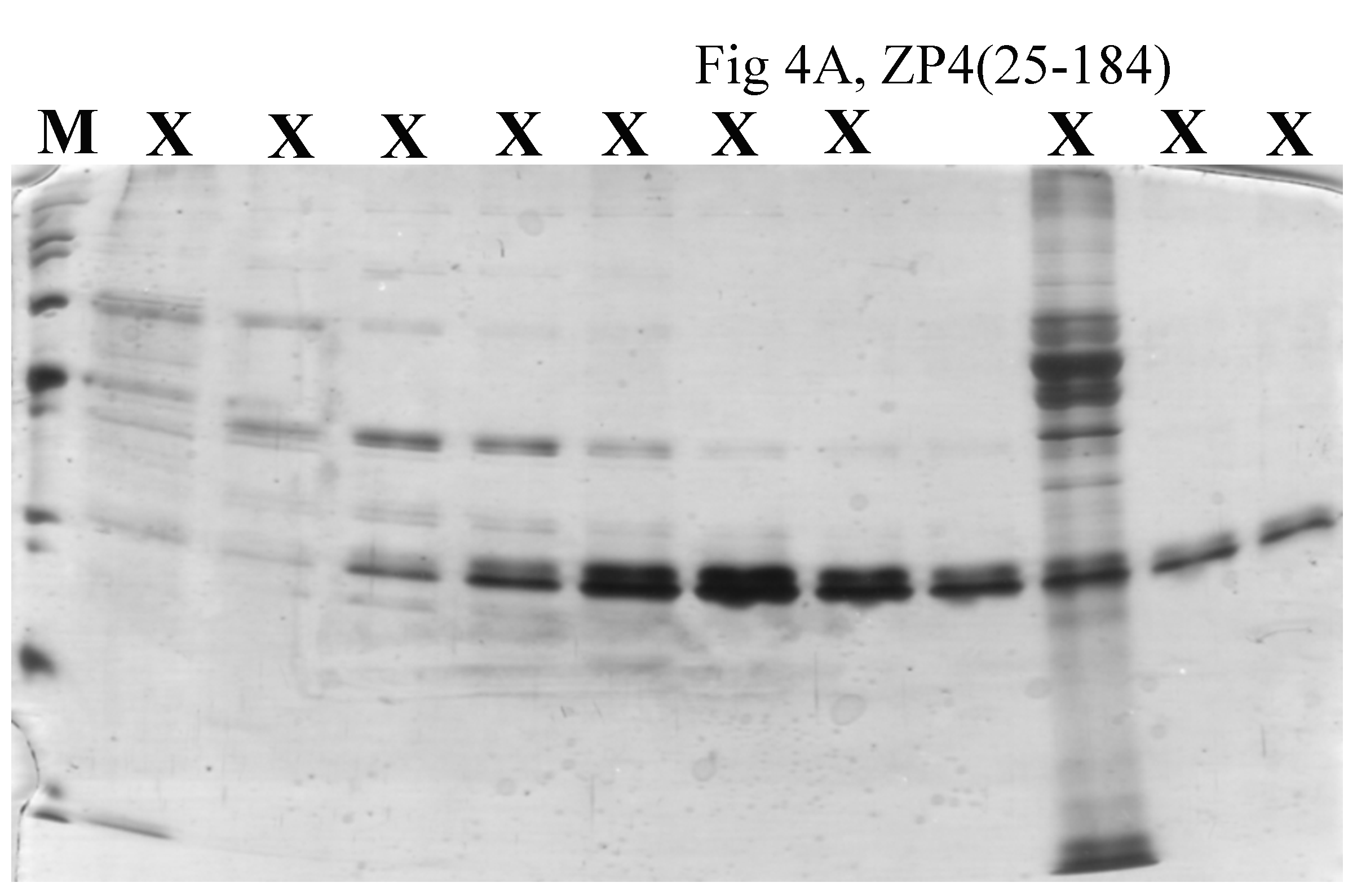

Supplement: S1 Fig — (ZIP) [file pone.0254234.s001.zip › S1 Fig 4A ZP4(25-184).TIF]

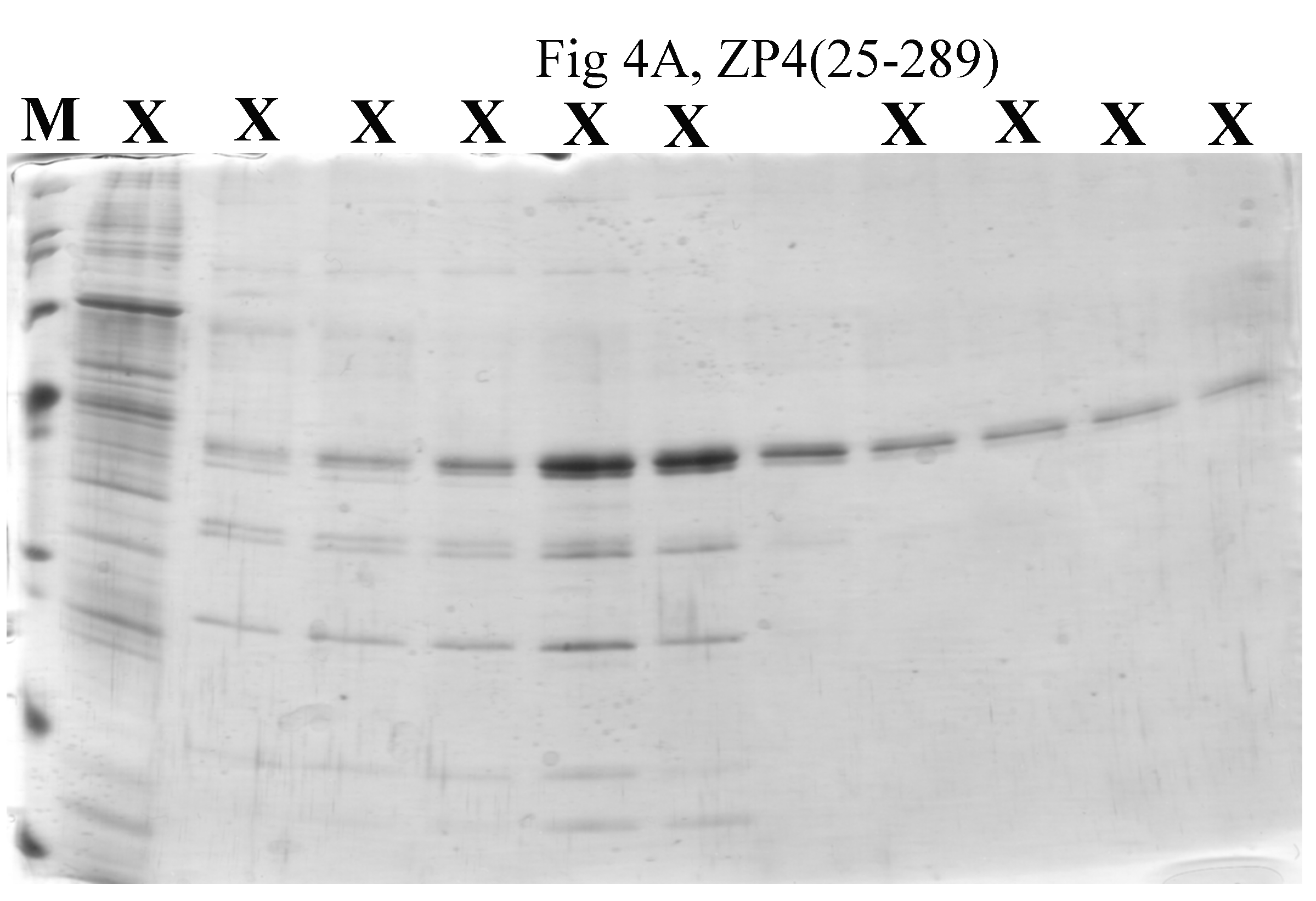

Supplement: S1 Fig — (ZIP) [file pone.0254234.s001.zip › S1 Fig 4A ZP4(25-289).TIF]

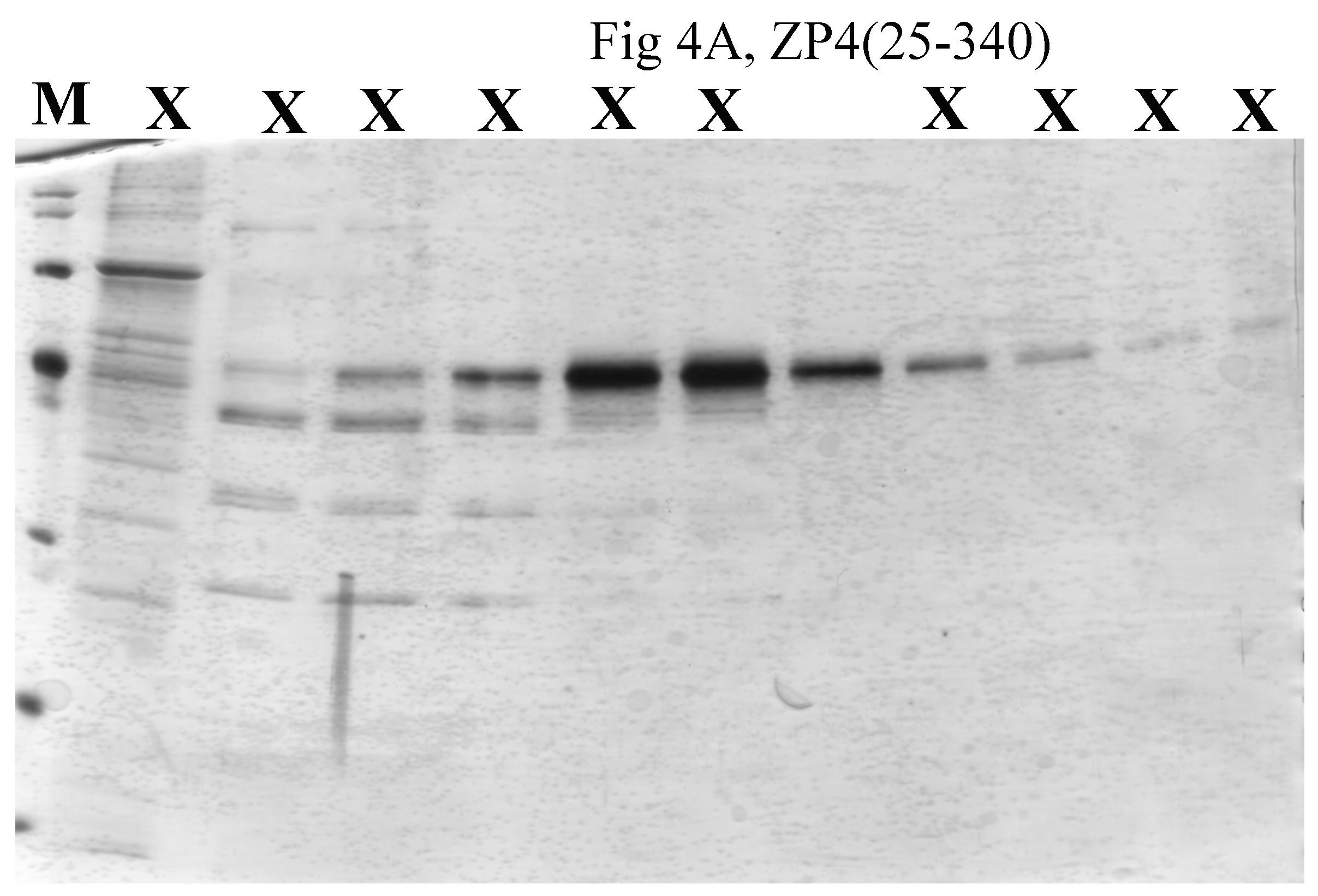

Supplement: S1 Fig — (ZIP) [file pone.0254234.s001.zip › S1 Fig 4A ZP4(25-340).TIF]

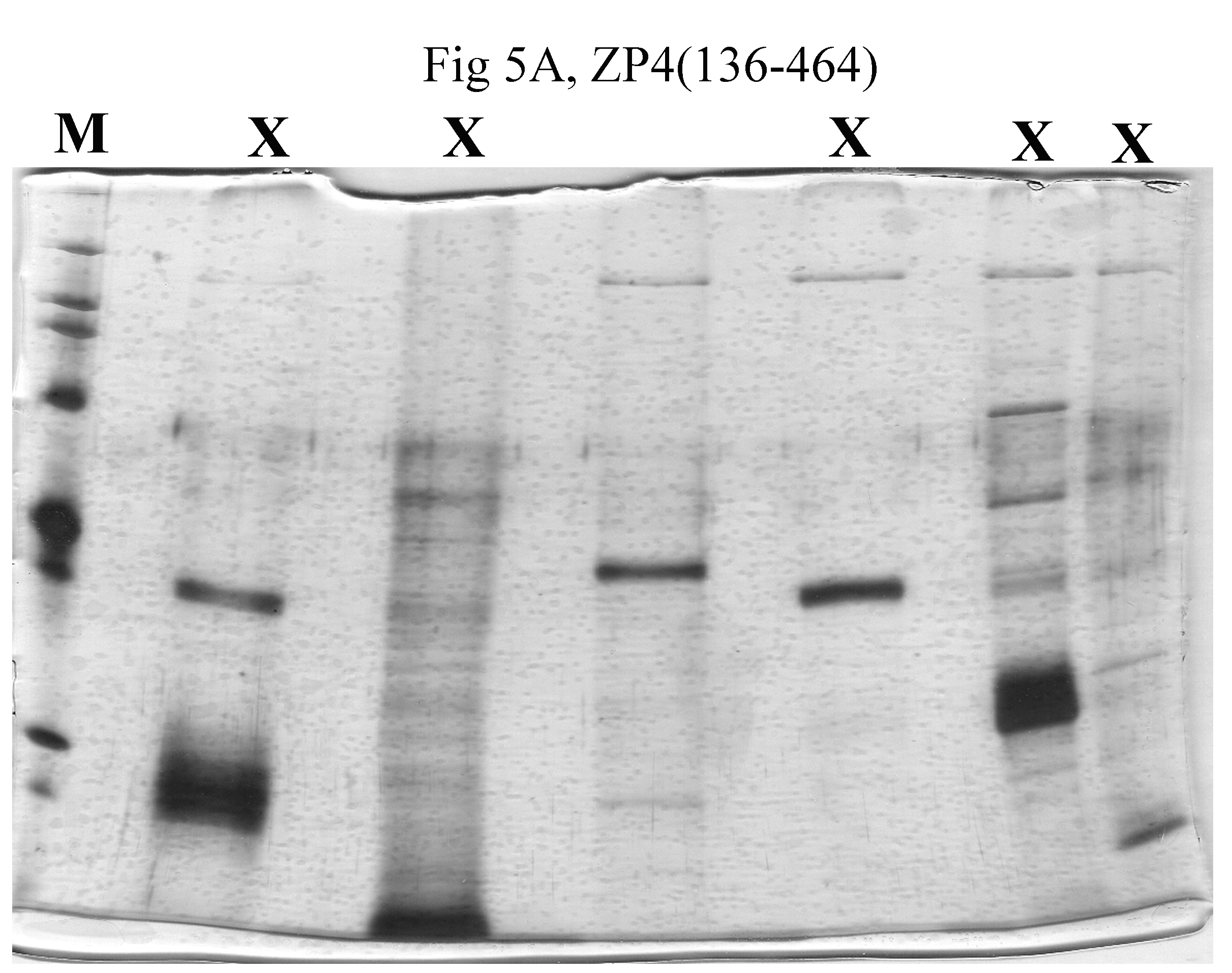

Supplement: S1 Fig — (ZIP) [file pone.0254234.s001.zip › S1 Fig 5A ZP4(136-464).TIF]

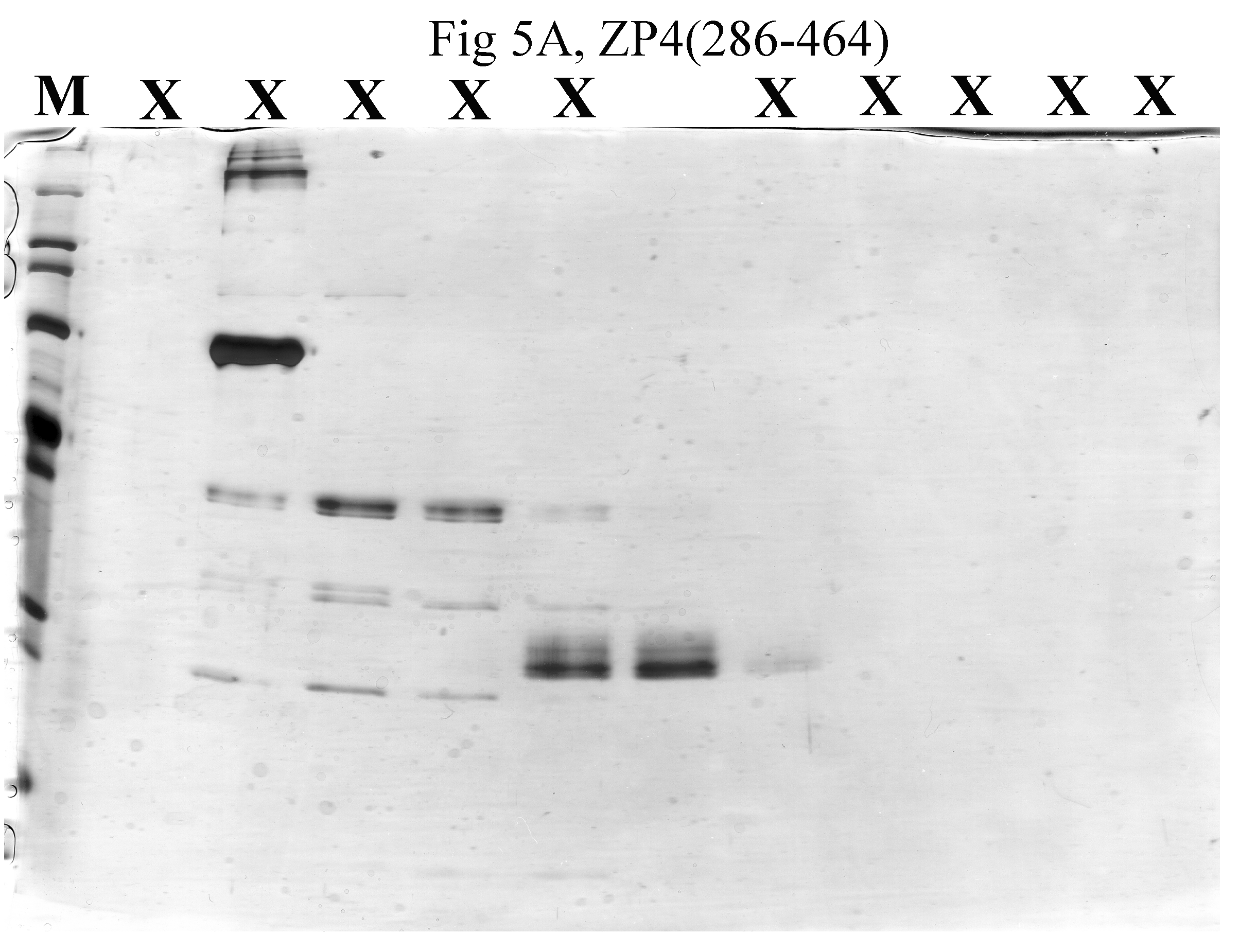

Supplement: S1 Fig — (ZIP) [file pone.0254234.s001.zip › S1 Fig 5A ZP4(286-464).TIF]

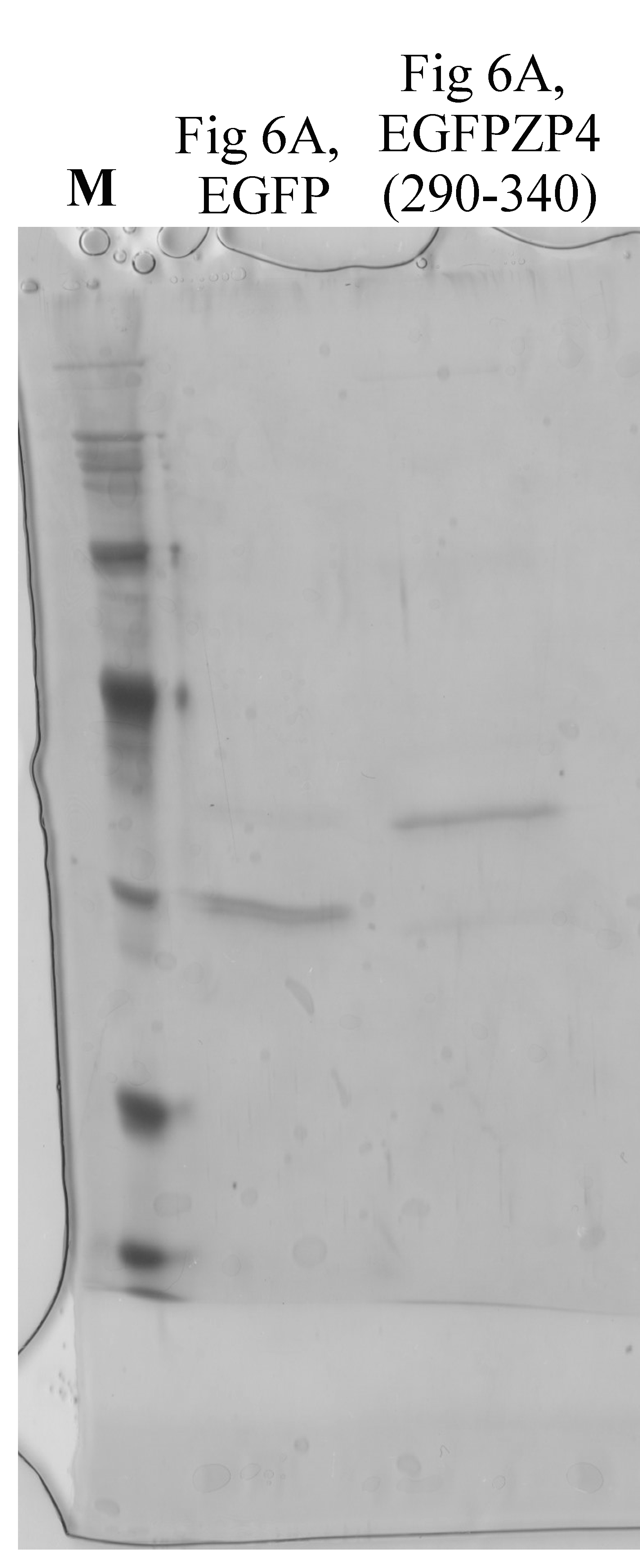

Supplement: S1 Fig — (ZIP) [file pone.0254234.s001.zip › S1 Fig 6A.TIF]
